# Development of a specific affinity-matured exosite inhibitor to MT1-MMP that efficiently inhibits tumor cell invasion *in vitro* and metastasis *in vivo*

**DOI:** 10.18632/oncotarget.7780

**Published:** 2016-02-27

**Authors:** Kenneth A. Botkjaer, Hang Fai Kwok, Mikkel G. Terp, Aneesh Karatt-Vellatt, Salvatore Santamaria, John McCafferty, Peter A. Andreasen, Yoshifumi Itoh, Henrik J. Ditzel, Gillian Murphy

**Affiliations:** ^1^ Department of Oncology, University of Cambridge, Cancer Research UK Cambridge Institute, Li Ka Shing Centre, Cambridge CB2 0RE, U.K; ^2^ Department of Cancer and Inflammation Research, Institute of Molecular Medicine, University of Southern Denmark, Odense, Denmark; ^3^ IONTAS Ltd., Babraham Research Campus, Cambridge CB22 3AT, U.K; ^4^ Kennedy Institute of Rheumatology, University of Oxford, Headington, Oxford OX3 7FY, U.K; ^5^ Department of Molecular Biology and Genetics, Aarhus University, Aarhus, Denmark; ^6^ Danish-Chinese Centre for Proteases and Cancer, Aarhus University, Aarhus, Denmark; ^7^ Department of Oncology, Odense University Hospital, Odense, Denmark; ^8^ Faculty of Health Sciences, University of Macau, Taipa, Macau SAR

**Keywords:** MT1-MMP, antibody, non-catalytic sites, in vivo targeting, metastasis

## Abstract

The membrane-associated matrix metalloproteinase-14, MT1-MMP, has been implicated in pericellular proteolysis with an important role in cellular invasion of collagenous tissues. It is substantially upregulated in various cancers and rheumatoid arthritis, and has been considered as a potential therapeutic target. Here, we report the identification of antibody fragments to MT1-MMP that potently and specifically inhibit its cell surface functions. Lead antibody clones displayed inhibitory activity towards pro-MMP-2 activation, collagen-film degradation and gelatin-film degradation, and were shown to bind to the MT1-MMP catalytic domain outside the active site cleft, inhibiting binding to triple helical collagen. Affinity maturation using CDR3 randomization created a second generation of antibody fragments with dissociation constants down to 0.11 nM, corresponding to an improved affinity of 332-fold with the ability to interfere with cell-surface MT1-MMP functions, displaying *IC*_50_ values down to 5 nM. Importantly, the new inhibitors were able to inhibit collagen invasion by tumor-cells *in vitro* and *in vivo* primary tumor growth and metastasis of MDA-MB-231 cells in a mouse orthotopic xenograft model. Herein is the first demonstration that an inhibitory antibody targeting sites outside the catalytic cleft of MT1-MMP can effectively abrogate its *in vivo* activity during tumorigenesis and metastasis.

## INTRODUCTION

Membrane-type 1 matrix metalloproteinase (MT1-MMP) is a type I transmembrane proteinase thought to be a major effector of pericellular proteolysis in a wide range of cell types [1]. The ability of MT1-MMP to cleave various extracellular matrix (ECM) components, including types I, II and III collagens, fibrin, and laminin 1 and 5, reflect its potential activities during cell invasion of ECM. Further, it can regulate cell signaling functions in response to the changing pericellular environment by the modulation of cell surface receptors, such as the αv and α5 integrins and the ectodomains of syndecan and CD44, as well as some growth factors. MT1-MMP can also initiate proteolytic cascades by activating other MMPs, including pro-MMP-2, pro-MMP-8 and pro-MMP-13 [2–4]. MT1- MMP over-expression has been detected in tumor cells and adjacent stromal cells in a variety of human tumors and has been correlated with poor clinical outcomes [5, 6]. Numerous studies of tumor cells and cancer-associated stromal cells have demonstrated an unequivocal role in the ability of a number of cell types to invade the ECM [7–9]. In angiogenesis, the fibrinolytic and collagenolytic activities of MT1-MMP are directed to the sprouting tip of endothelial cells as they invade the surrounding ECM [10, 11]. In a 3D tumor model, we showed that MT1-MMP activity was required for neo-angiogenesis not only by endothelial cells, but also by associated fibroblasts [12]. The pro-angiogenic effects of MT1- MMP have additionally been ascribed to up-regulation of VEGF-A via activation of a Src/Akt kinase-dependent signaling pathway [13]. MT1-MMP is an effector of synovial pannus invasion and destruction of cartilage in Rheumatoid Arthritis [14]. It drives the migration and function of autoreactive T-cells [15] and of macrophages in peripheral nerve injury [16], suggesting that this pericellular protease plays significant role in a number of pathological processes and should be considered a viable target for therapeutic interventions.

It is broadly known that therapeutic targeting of members of the MMP family demands greater specificity of MMP inhibitors. The recent development of newer generations of more specific inhibitors [17] has demonstrated that MMPs that are more unambiguously implicated in pathological processes could indeed be good therapeutic targets. However, small molecule inhibitors directed at the highly homologous active sites of MMPs are inevitably less specific, and the concept of targeting extra-catalytic motifs determining substrate specificity may be a useful approach [18]. Therapies with human recombinant antibodies also represent a significant alternative to small molecule-based targeted therapies in cancer treatment [19]. MT1-MMP has been a successful target in this respect [20], and a number of antibodies targeting extra catalytic sites of this protease have been described [21–23]. We have refined this concept using antibody maturation techniques via CDR3 randomization and re-selection to fine-tune the isolation of potent antibody derivatives against MT1-MMP exosites. Furthermore, since there is a notable discrepancy in the *in vitro* efficacy of inhibitors against a soluble form of the enzyme and the activity at the surface of cells, we have utilized cell-based screening techniques to isolate inhibitors that demonstrate substantial *in vivo* efficacy.

## RESULTS

### Isolation of scFv antibodies inhibiting MT1-MMP

**Figure 1:**
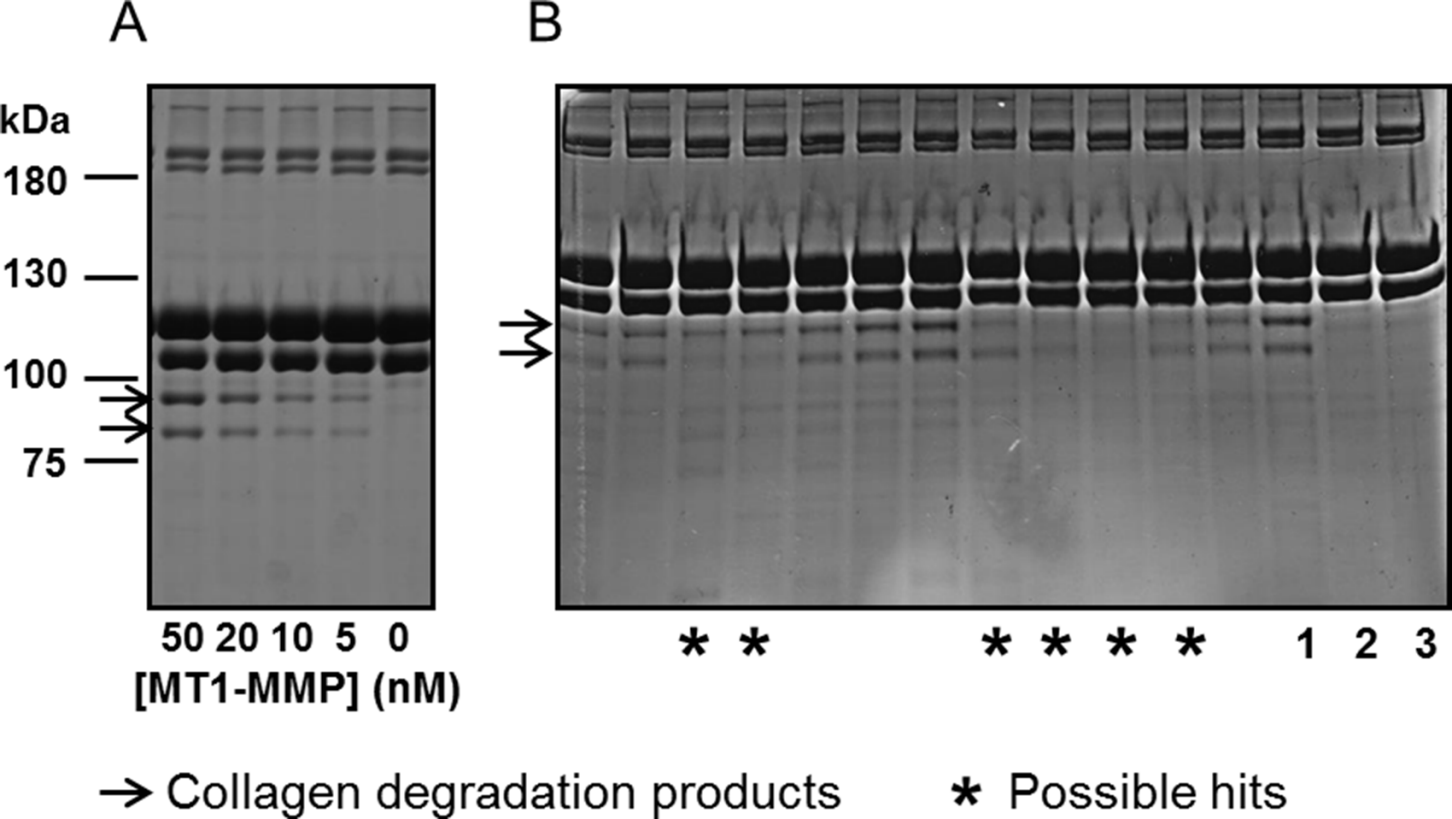
MT1-MMP scFv's selected for the ability to prevent collagen cleavage (**A**) Aliquots of ectodomain MT1-MMP (50, 20, 10, 5 or 0 nM) were incubated with fibrillated collagen type I for 16 hours at room temperature. The reaction products were analyzed by reducing SDS-PAGE. Collagen cleavage was observed as the conversion of the M_r_ ∼100 kDa bands of collagen type I to the M_r_∼75 kDa ¾-fragments. (**B**) In separate reactions, ectodomain MT1-MMP was pre-incubated with *E.coli*. supernatant containing scFv-antibodies selected to MT1-MMP. As indicated by stars, some scFv's were identified as possible hits for interfering with MT1-MMP catalyzed collagen cleavage. In lanes indicated by numbers, MT1-MMP was pre-incubated with control Fc-scFv DES (1), with CT1746 (2) or sample without MT1-MMP (3).

**Figure 2:**
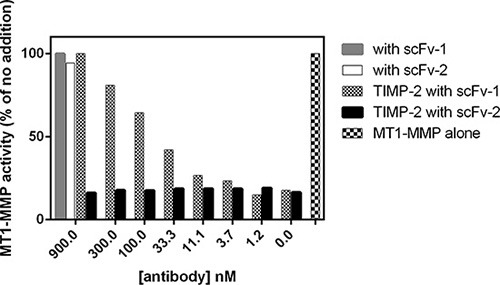
MT1-MMP scFv's selected for the ability to interfere with interactions to TIMP-2 Selected scFv's were screened for the ability to compete with TIMP-2 for MT1-MMP binding. The activity of ectodomain MT1-MMP towards a small fluorogenic peptide substrate was inhibited by TIMP-2, but not by selected scFv's (scFv 1 and 2). However, the TIMP-2 inhibition could be competed away by some scFv's (scFv-1), but not by others (scFv-2), as indicated in the figure.

With the aim of generating specific potent antibodies capable of inhibiting MT1-MMP function at the cell-surface, we chose to target epitopes outside the highly conserved catalytic cleft. The recombinant full ectodomain of pro-MT1-MMP E240A, which lacks proteolytic activity due to the substitution of the active site glutamate with alanine, was used as the target antigen for selection of binding scFvs from a naïve human scFv phage-display library [24]. N-terminal sequencing confirmed that the pro-domain of recombinant pro-MT1-MMP E240A was still present and therefore occluding the catalytic cleft. Solution phase selection was carried out using biotinylated pro-MT1-MMP E240A antigen and streptavidin-coated agarose beads, which thus pulled down antibody fragment clones binding to pro-MT1-MMP. Clones chosen for further analysis were selected based on the presence of unique heavy and light chain CDR3 sequences and strong binding in ELISA. MT1-MMP inhibitory properties of selected clones were evaluated using a macromolecular substrate cleavage assay. Fibrillar collagen type I was incubated with the active form of recombinant wt MT1- MMP in the presence or absence of purified scFv's and the cleavage products analyzed using SDS-PAGE. As shown in Figure 1A, collagen is cleaved progressively with increasing MT1-MMP concentration. In the presence of individual scFv's clones, collagen cleavage was inhibited to varying degrees up to 100% inhibition (Figure 1B). As controls, the general hydroxamate-based MMP inhibitor CT1746 showed full inhibition, whereas a negative control antibody fragment (against intracellular desmin; Fc- scFv DES) exhibited no inhibition. Selected MT1- MMP binding scFv's were further characterized using a fluorogenic peptide substrate assay. As shown in Figure 2, addition of scFv's did not inhibit MT1-MMP activity in the absence of TIMP-2, confirming that selected scFv's bind to MT1-MMP outside the catalytic cleft. Some scFv's were found to inhibit the binding of TIMP-2 to the active MT1-MMP. MT1-MMP activity was almost completely inhibited in the presence of 10-fold excess TIMP-2 in the absence of scFv's, but pre-incubation of MT1-MMP with several scFv's protected MT1-MMP from TIMP-2 inhibition (exemplified by scFv-1, Figure 2) The same scFv's outcompeted the N-terminal fragment of TIMP-3 (N-TIMP-3) from binding to MT1-MMP, showing that it was the N-terminal part of the TIMP molecule that was competed away (data not shown). We thus screened scFv antibodies using these two macromolecular screening approaches and narrowed down the initially selected scFv's to 27 inhibitory clones.

### Assessment of functional properties of lead antibodies using assays dependent on cell-membrane bound MT1-MMP activity

**Figure 3:**
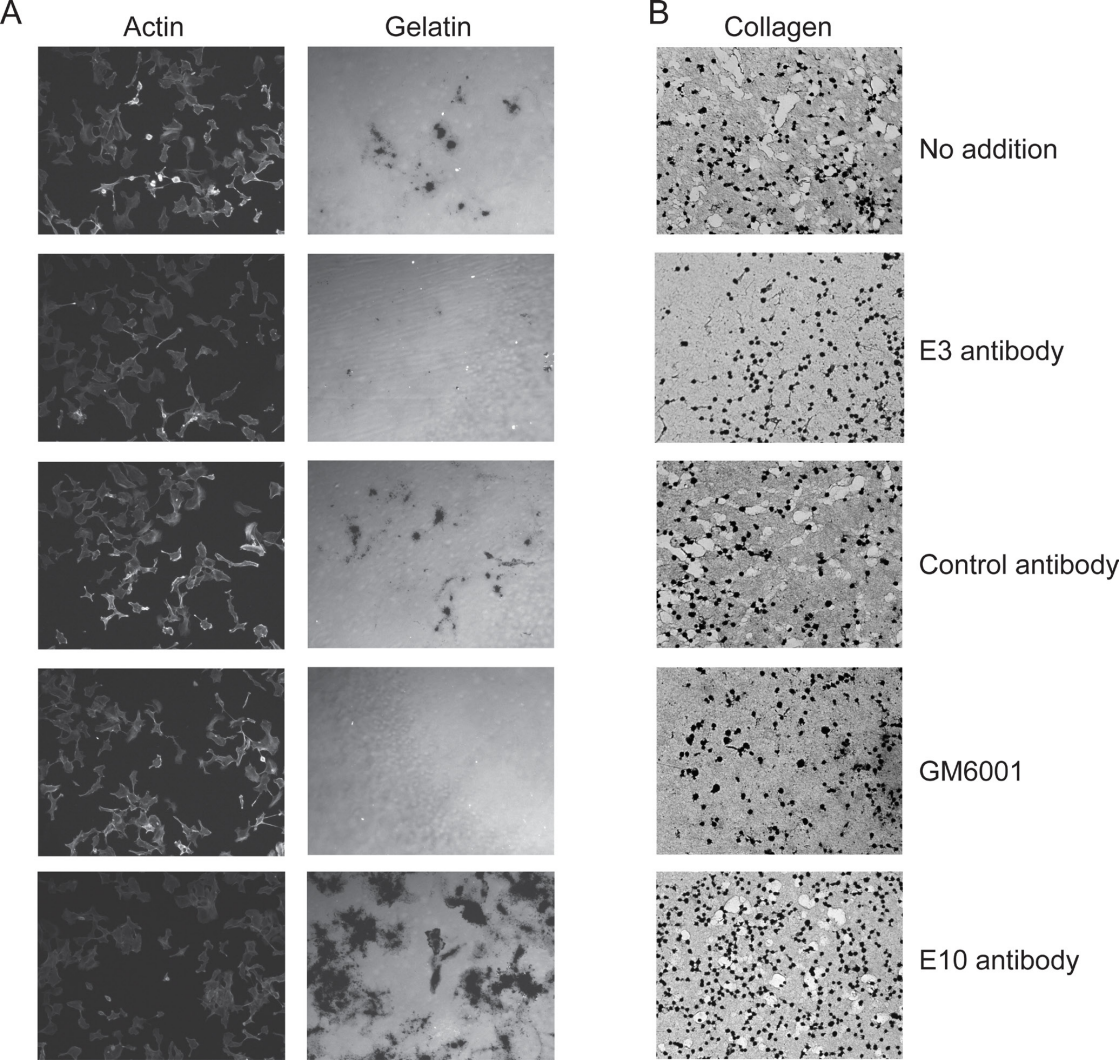
Lead Fc-scFv's inhibit cell-surface MT1-MMP gelatinolytic and collagenolytic activity HT-1080 cells were cultured on fluorescently-labeled gelatin (**A**) or collagen film (**B**) in the absence or presence of purified MT1-MMP Fc-scFv's, DES control or GM6001. For gelatinolytic activity, cells were fixed after 18 hours and imaged. Staining of actin ensured similar cell numbers in areas of imaging. For collagenolytic activity, cells were fixed after 3 days of incubation and collagen stained with Coomasie Brilliant Blue. As exemplified by Fc-scFv E3, a few clones were able to inhibit both gelatin and collagen degradation. Control Fc-scFv DES served as negative control, whereas GM6001 gave complete inhibition (A and B).

**Figure 4:**
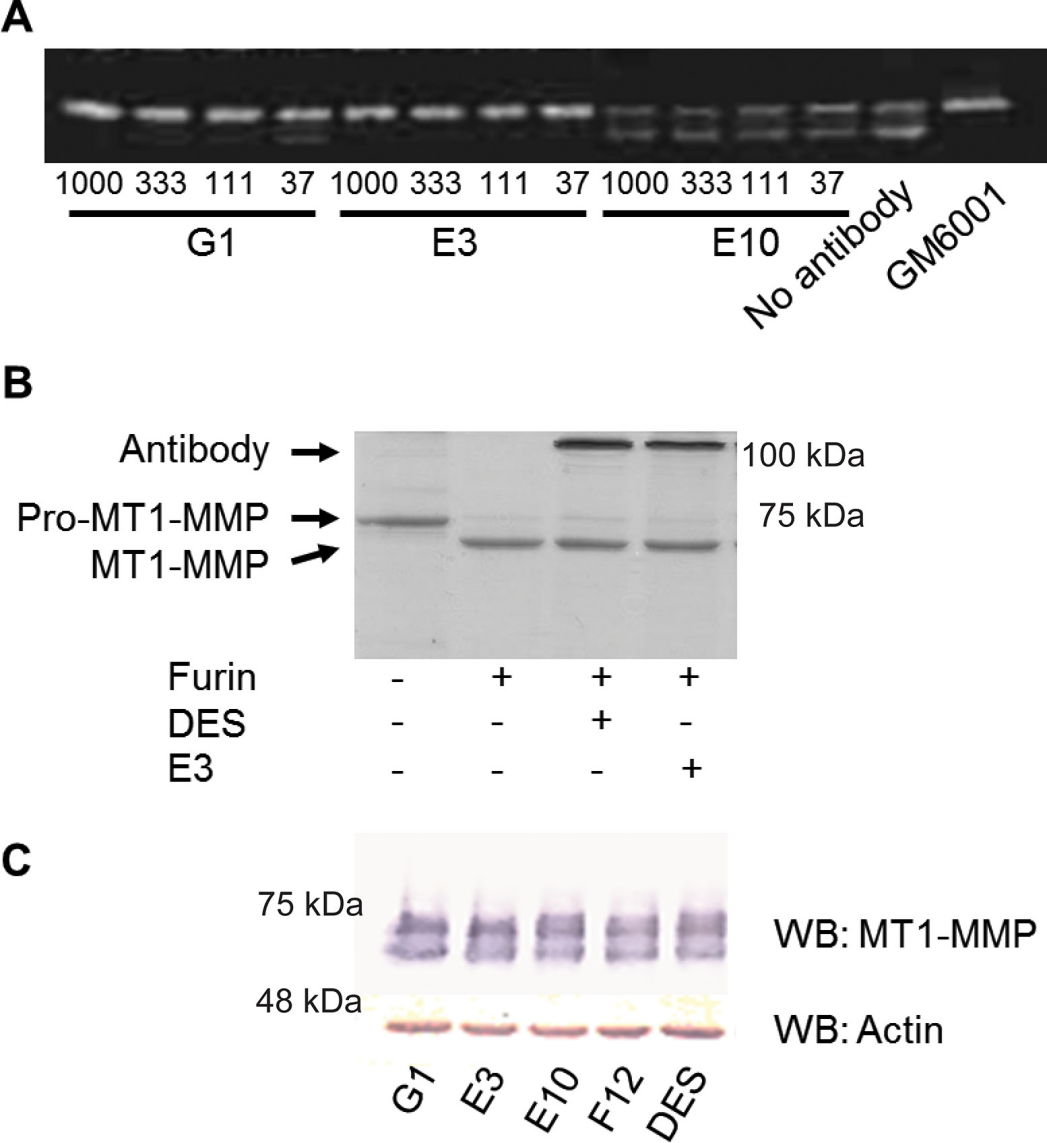
Fc-scFv's inhibit activation of pro-MMP-2, but not activation of pro-MT1-MMP (**A**) For pro-MMP-2 activation assays, HT-1080 cells were cultured in the presence of trace amounts of collagen type I in the absence or presence of MT1-MMP Fc-scFv's or GM6001. Following 18 hours of incubation, cell culture supernatant was analysed for levels of pro-MMP-2 and active MMP-2 by zymography. A few lead Fc-scFv's displayed inhibitory activity as shown by E3 and G1, while the majority did not, as exemplified by E10. The experiments shown are representative of a total of three independent experiments. (**B**) Full ectodomain Pro-MT1-MMP was incubated with or without furin, and in the presence or absence of DES Fc-scFv control or E3, as indicated, for 3 hours at RT. Reaction products were analyzed by reducing SDS-PAGE, and conversion of pro-MT1-MMP to MT1-MMP by furin was observed. (**C**) Analysis of HT1080 cell lysates for the presence of MT1-MMP was carried out by Western blotting using anti-MT1-MMP polyclonal antibody N175/6. As a loading control, actin was detected by rabbit anti-actin polyclonal antibody.

To identify scFv antibodies inhibiting the catalytic activity of cell-surface MT1-MMP, we reformatted them into Fc-scFv formats for improved stability and analyzed their ability to interfere with endogenously expressed MT1-MMP on HT-1080 cells. First, the effect on degradation of fluorescent-labeled gelatin (F-gelatin) films by HT-1080 cells was examined (Figure 3A, left panel) and we confirmed that gelatin film degradation activity in HT1080 cells is due to MT1-MMP activity (data not shown). As a result, three clones, E3, G1 and C2, were found to inhibit fluorescent-labeled gelatin film degradation, with the most potent being E3 (Figure 3A, right panel). Many of the lead Fc-scFv's were not inhibitory, however two antibodies strongly stimulated gelatin film degradation (E10, Figure 3A). A broad- spectrum hydroxamate-based MMP inhibitor, GM6001, completely inhibited gelatin-film degradation, whereas control Fc-scFv DES showed no effect (Figure 3A, right panel, GM6001 acts in a similar manner to CT1746 and the two were used interchangeably). Next, the effect of lead Fc-scFv antibodies on cell-surface collagenolytic activity of MT1-MMP was evaluated using HT-1080 cells. GM6001 completely inhibited collagen degradation by endogenous MT1-MMP. Fc- scFv E3, G1 and C2 were inhibitory, with E3 showing the most potent inhibition of collagenolytic activity (Figure 3B). E10 Fc- scFv did not show noticeable effect on collagen degrading activity (Figure 3B). Next, the effect of Fc-scFv's on cell surface pro-MMP-2 activation was examined. HT-1080 cells were cultured in the presence of Fc-scFv's and culture media were analyzed by gelatin zymography. As shown in Figure 4A, the Fc-scFv antibodies E3 and G1 potently inhibited pro-MMP-2 activation, while other clones showed no inhibitory activity. E10 had no effect on pro-MMP-2 activation, and GM6001 completely inhibited the activation. Since Fc-scFv E3 and G1 could also compete with TIMP-2 binding to MT1-MMP, the inhibitory mechanism of pro-MMP-2 activation might be dual; displacing TIMP-2 from cell-surface MT1-MMP that would normally serve as receptor for bringing pro-MMP-2 to active free MT1-MMP and/or sterically hindering interactions between free MT1- MMP and its macromolecular substrate pro-MMP-2. In conclusion, two Fc-scFv antibodies, E3 and G1, displayed inhibitory activity towards all three cell-surface MT1-MMP functions whereas Fc-scFv C2 showed a minor effect on gelatinolytic and collagenolytic MT1-MMP activity.

### Lead Fc-scFv antibodies display orthosteric inhibition by binding to the MT1-MMP catalytic domain outside the catalytic cleft

Given that E3 showed the most potent inhibition of cell-surface MT1-MMP, we investigated the mechanism behind this in greater detail. Initially, we evaluated the effect of E3 on the activation of pro-MT1-MMP (containing the pro-domain) by furin. Recombinant pro-MT1-MMP could be activated by furin over time, as visualized on SDS-PAGE. However, in the presence of Fc-scFv E3, no delay of pro-MT1-MMP activation by furin could be observed (Figure 4B), indicating that the observed functional effect of E3 was not due to inhibition of pro-MT1-MMP activation cleavage. Also, western blotting analysis of MT1-MMP from cell lysates of Fc-scFv E3 treated HT-1080 cells did not reveal any differences in MT1-MMP in terms of the amount of enzyme or its activation status compared to other selected antibodies or negative antibody control treatment (Figure 4C). We next examined the E3 binding site of MT1-MMP using recombinant forms of the individual domains. The binding affinity (*K*_D_) of Fc-scFv E3 for the isolated catalytic domain of MT1-MMP was determined to be 22.4 ± 14.5 nM using surface plasmon resonance (SPR) analysis, which is comparable to that of the ectodomain (*K*_D_ 36.5 ± 5.3 nM), whereas no binding to the isolated hemopexin domain was observed. In addition, pre-incubating 20 nM MT1-MMP catalytic domain with 250 nM TIMP-2 abolished binding to E3, whereas pre-incubating MT1-MMP with the active site-oriented small molecule hydroxamate inhibitor, CT1746, did not (data not shown).

**Figure 5:**
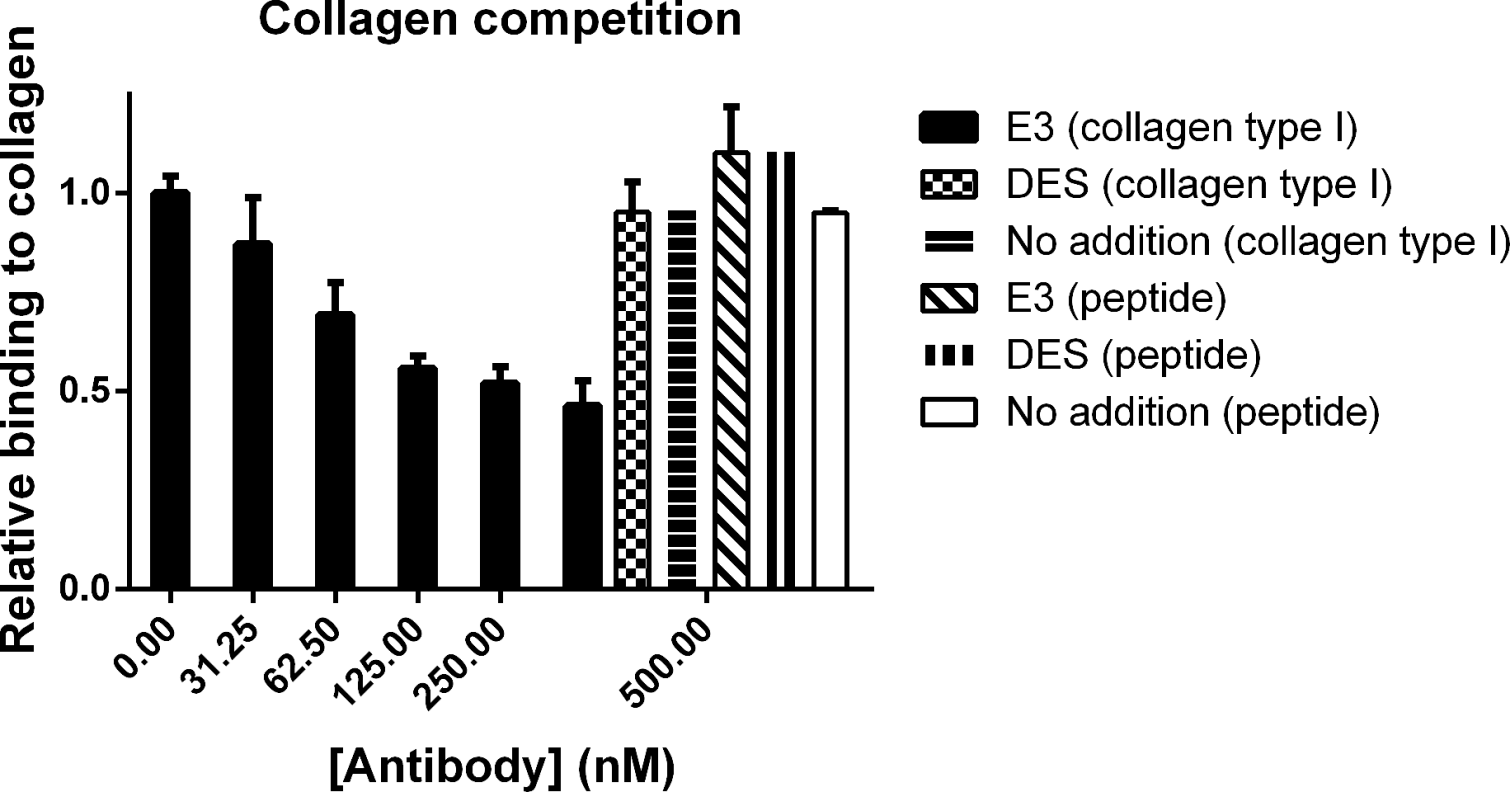
Fc-scFv's compete with collagen type I for binding to MT1-MMP whereas binding of collagen peptide is unaffected In an ELISA setup, pepsin-extracted collagen type I or collagen peptide were coated in wells after which ectodomain MT1- MMP was allowed to bind in the absence or presence of Fc-scFv E3 or DES control antibody at a concentration of 0-500 nM.

Next, we investigated the effect of E3 on the ability of MT1-MMP to bind type I collagen. The data indicated that the binding of MT1-MMP to the collagen was decreased by Fc-scFv E3 in a dose-dependent manner. Interestingly, when a triple helical collagen peptide spanning 7–17′ of the collagen II cleavage site [25, 26] was used instead of full length collagen type I, the binding of MT1-MMP was not outcompeted (Figure 5). Thus, ligands binding directly in the active site, such as the hydroxamate inhibitor, CT1746 or the small fluorogenic peptide substrate, or likely the collagen peptide, are not affected by E3, whereas binding of larger ligands such as collagen type I and TIMP-2 is abrogated. We thus concluded that the Fc-scFv E3 is an orthosteric antagonist since it competes with the MT1-MMP interaction with macromolecular substrates.

### Lead Fc-scFv antibodies are specific for MT1-MMP

We next examined the specificity of the E3 and G1 Fc-scFv antibodies using a TIMP-competition assay in which a number of proteinases were assayed by hydrolysis of a small fluorogenic peptide substrate in the presence of either TIMP-2 or TIMP-3. When using either the ectodomain or the catalytic domain of MT1- MMP, the inhibition observed by both TIMP-2 and TIMP-3 was reversed by the Fc-scFv E3 and G1, but not by control DES (Figure 2 and Supplementary Figure 1). We confirmed that no such effect of the Fc-scFv's was observed when using MT2-MMP, MT3-MMP, MT5-MMP, MMP-1, MMP-2, MMP-3, MMP-7, MMP-9, MMP-10, MMP-13, MMP-19, MMP-26 and ADAM17 inhibited by either TIMP-2 or -3 (Supplementary Figure 1).

### Improving lead Fc-scFv's antibodies to generate variants with high binding affinity and inhibitory potency

Given that Fc-scFv E3 and G1 displayed inhibitory activity against several activities of cell-surface MT1-MMP, we decided to further improve their affinity using an affinity maturation technique. For each of the two Fc-scFv's, new phage-display libraries were prepared focusing on the CDR3 loop of both the heavy and light chains, since these loops usually are central to antigen binding. Kunkel mutagenesis was employed to obtain variant antibody fragments, with blocks of 5–6 amino acid residues in the CDR3 loops being randomized to yield a total of 5 and 6 new phage libraries for E3 and G1, respectively, and with a diversity of up to 6.4 × 10^7^ amino acid combinations for each new library. In affinity selections, light and heavy chain libraries were kept separate, and the concentration of pro-MT1-MMP E240A antigen was continuously decreased from 1 nM down to 5 pM. Subsequently, an off-rate selection round was carried out to select antibody scFv-fragments with the slowest off-rates. Thus, biotinylated MT1-MMP antigen was allowed to bind phage display selection outputs from affinity selections before a large excess of non-biotinylated MT1-MMP was added. Following incubations of up to 20 h, phage-displayed antibody fragments still binding biotinylated-MT1-MMP were pulled down.

**Figure 6:**
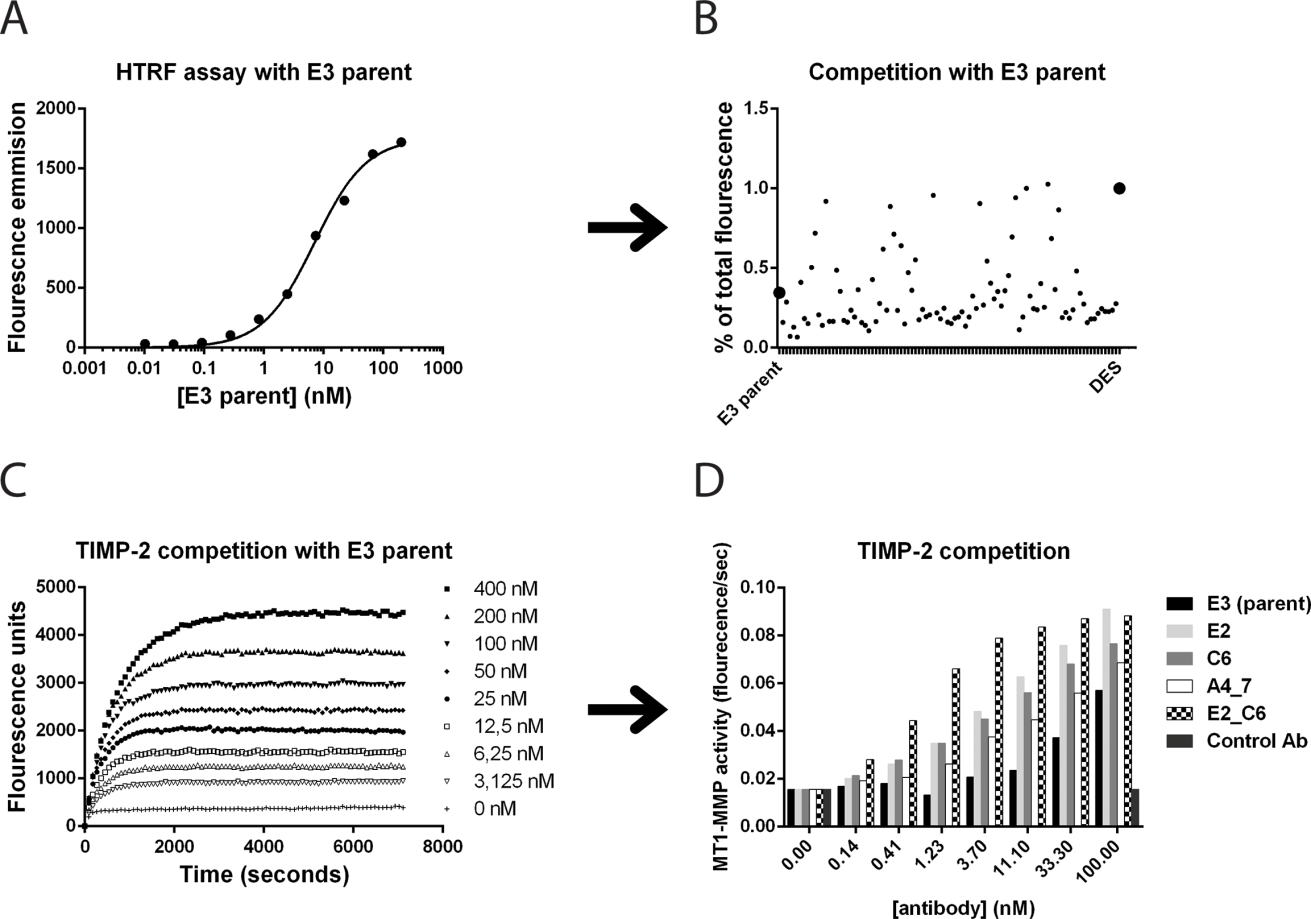
Affinity maturation of lead antibody clones and screening for improved variants Homogeneous time-resolved fluorescence (HTRF), TIMP-2 competition and surface plasmon resonance assays were utilized to identify affinity improved variants of lead Fc-scFv's. (**A**) In HTRF assays, parent E3 binding to MT1-MMP was observed by direct labeling of E3 and detection with labeled streptavidin and biotinylated full ectodomain pro-MT1-MMP. (**B**) The ability to outcompete labeled parent Fc-scFv E3 from binding to MT1-MMP was detected as a decrease in HTRF signal and displayed as percent of total fluorescence in the absence of competing scFv. (**C**) Cleavage of small fluorogenic peptide by ectodomain MT1-MMP was inhibited by TIMP-2. E3 Fc-scFv E3 outcompeted TIMP-2 inhibition of MT1-MMP, as shown by increasing E3 concentrations from 0–400 nM. (**D**) The efficacy of improved variants to outcompete TIMP-2 from ectodomain MT1-MMP was directly compared to E3 parent.

To retain the original epitope binding sites as the parent E3 and G1 in the new antibody variants, a homogenous time-resolved fluorescence (HTRF) assay was used to identify improved scFv clones in which competition of new clones with parent Fc-scFv E3 was measured. The parent E3 was used as reference in all subsequent analysis of improved variants. As shown in Figure 6A, a HTRF binding curve of Fc-scFv E3 parent to MT1-MMP was obtained by labelling E3 with a d2-acceptor dye and using biotinylated MT1-MMP together with a streptavidin europium-cryptate donor. A competition assay was set up based on the specific E3 binding curve and used to monitor displacement of d2-labeled E3 parent by the new affinity-improved scFv-antibodies directly from *E. coli.* cultures (Figure 6B). In parallel, the TIMP-2 competition assay was again used to identify antibody variants capable of competing with TIMP-2 for MT1-MMP binding. Based on these two assays, lead affinity-matured clones were selected from 1152 screened clones, reformatted into Fc-scFv, expressed and purified, and tested in the TIMP-2 competition assay with increasing Fc-scFv concentrations (Figure 6C and 6D). A number of identified clones showed increased TIMP-2 competition compared to parent E3, whereas the negative control Fc- scFv DES showed no competition (Figure 6C and 6D). Importantly, the affinity improved Fc-scFv variants showed no binding to homologous proteases when analysed in the same TIMP competition assay as above for parent E3 and G1 (data not shown).

### Kinetics of affinity matured MT1-MMP Fc-scFv's

To directly evaluate whether the 2nd generation E3 and G1 clones possessed an increased affinity to MT1- MMP, SPR was employed using the Fc-scFv format of each antibody. New variants showed both faster association and slower dissociation rates, and Table I summarizes the binding kinetics for the new variants compared to parent E3 and G1. The increase in binding affinity was up to 20- and 70-fold for E3 and G1 variants, respectively, with the highest binding clones being C6 (*K*_D_ 1.88 nM) and E2 (*K*_D_ 0.92 nM). In addition, Fc-scFv E2 was immobilized directly to a sensor chip and used for competition analysis by measuring the binding to MT1-MMP alone or to MT1-MMP pre-complexed with antibodies. As expected, parent E3 competed with the MT1-MMP binding (data not shown).

From the sequencing of 384 new variant clones selected from activity screening, it was evident that for the G1 lineage, the best performing variants were almost exclusively changed in the heavy chain CDR3 (Table 1). The same trend was found for the E3 lineage, however more variant clones from this parent were found to be changed in the light chain CDR3, such as the highest affinity clone C6. The light chain of C6 was therefore combined with different heavy chain variants of E3 and with the heavy chain of the best G1 lineage clone E2. Surprisingly, the combination between the C6 light chain and the E2 heavy chain, named E2_C6, was functional and clearly more active than each of the variants alone, showing a *K*_D_ of 0.11 nM, corresponding to a 332-fold increased binding affinity compared to the E3 parent (Figure 6D and Table I). Interestingly, the two cysteines present in the G1 parent heavy chain CDR3 were conserved in all new G1 variants, indicating that these two residues are required to preserve binding affinity, potentially forming a closed loop structure of the CDR3.

**Table 1:** Sequence and kinetic binding constants for selected anti-MT1-MMP Fc-scFv's

Clone	CDR3 heavy chain	CDR3 light chain	*k*_on_ (×10^4^) (M^−1^s^−1^)	*k*_off_ (×10^−4^) (s^−1^)	*K*_D_ (nM)	*K*_D_(parent)/*K*_D_(new)
E3 parent	FAGREGESDI	QQSYSTPST	2.34 ± 0.51	8.4 ± 0.9	36.5 ± 5.3	1
A2	FWGNLGESDI	QQSYSTPST	1.50 ± 0.71	2.3 ± 0.2	16.6 ± 6.4	2.2
A4_7	FSPPPGESDI	QQSYSTPST	13.4 ± 3.05	5.3 ± 0.7	3.99 ± 0.4	9.15
A6_5	FAVYQPWEDI	QQSYSTPST	18.6 ± 14.5	4.0 ± 2.4	2.34 ±0.6	15.6
A6_2	FLPPPGESDI	QQSYSTPST	43.3 ± 54.1	7.2 ± 8.2	2.21 ± 0.9	16.5
C6	FAGREGESDI	LFPLSDPST	26.9 ± 26.7	3.0 ± 0.7	1.88 ± 1.1	19.4
G1 parent	IGYCSGGSCSSGDYGMDV	QQYNSYPLT	0.76 ± 0.51	3.5 ± 0.2	65.1 ± 48.4	1
E1	YCPGEAGSCSSGDYGMDV	QQYNSYPLT	0.34 ± 0.01	1.9 ± 0.03	56.5 ± 0.8	1.15
F1	YCPVEAGSCSSGDYGMDV	QQYNSYPLT	4.47 ± 1.90	1.2 ± 0.4	3.24 ± 2.3	20.1
E6	FCPFSQGSCSSGDYGMDV	QQYNSYPLT	21.8 ± 22.8	1.0 ± 0.33	1.12 ± 1.0	58.1
E2	FCPLSTGSCSSGDYGMDV	QQYNSYPLT	32.9 ± 11.8	3.2 ± 1.9	0.92 ± 0.2	70.8
E2_C6	FCPLSTGSCSSGDYGMDV	LFPLSDPST	18.8 ± 7.95	0.2 ± 0.1	0.11 ± 0.04	332 and 592

Amino acid sequence of CDR3 loops for selected anti-MT1-MMP antibodies are shown. Surface plasmon resonance (SPR) was used for kinetic analysis of the binding of selected Fc-scFv's to pro-MT1-MMP E240A. The *k*_on_, *k*_off_ and *K*_D_ values were determined by global fitting to a 1:1 binding model with the BIACORE evaluation program. *K*_D_ (parent)/*K*_D_ (new) is the fold improvement in *K*_D_ of the affinity matured variants compared to E3 and G1 respectively.

### Effects of improved Fc-scFv's on cell-surface MT1-MMP activities

**Figure 7:**
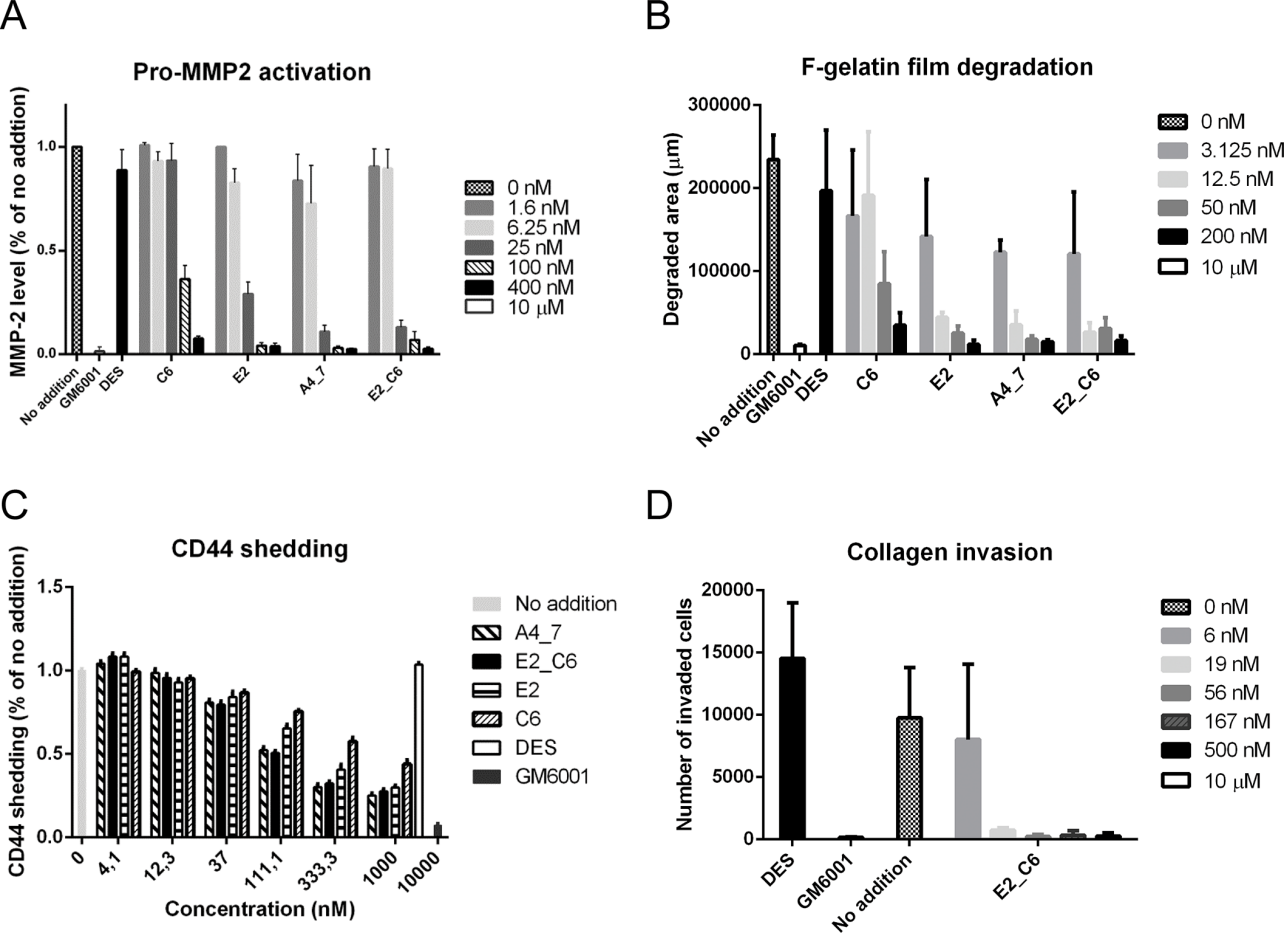
Affinity-improved Fc-scFv's effectively inhibit cell-surface MT1-MMP activity (**A**) HT-1080 cells were cultured in the presence of trace amounts of collagen type I, and in the absence or presence of MT1-MMP Fc-scFv's (0–400 nM), DES control (400 nM) or GM6001 (10 μM). Following 18 hours of incubation, cell culture supernatant was analyzed for levels of pro-MMP-2 and active MMP-2 by zymography. Quantification of active MMP-2 levels was done by densitometric analysis of inverted images. (**B**) HT-1080 cells were cultured on fluorescent-labeled gelatin in the absence or presence of MT1-MMP Fc-scFv's (0–200 nM), DES control Fc-scFv DES (200 nM) or GM6001 (10 μM). Cells were fixed after 18 hours and imaged. Quantification of gelatin degradation was done by densitometric analysis of inverted images. (**C**) HTC-75 cells were cultured in the absence or presence of MT1-MMP Fc-scFv's (0–1000 nM), DES control (1000 nM) or GM6001 (10000 nM). Following 15 hours of incubation, cell culture supernatant was analyzed for levels of CD44 shedding by ELISA. (**D**) Effect of Fc-scFv E2-C6 (0–500 nM), Fc-scFv DES control (500 nM) or GM6001 (10 μM) in a collagen invasion assay in which HT-1080 cells were allowed to invade through the collagen matrix for 24 hours towards cell medium containing fetal calf serum as attractant.

Since fluorescent-labeled gelatin film degradation and pro-MMP-2 activation assays were readily quantifiable, we used these two assays to directly evaluate the inhibitory potential of the improved Fc-scFv antibodies. As shown in Figure 7A and 7B, improved variants A4_7 (E3 lineage) and E2_C6 (mixed E3 and G1 lineage) showed the highest potency, with *IC*_50_ values between 3 nM and 25 nM in the two assays. The general MMP inhibitor CT1746 showed complete inhibition whereas no inhibition was observed with control Fc-scFv DES (Figure 7A and 7B). In addition, fluorescent-labeled gelatin degradation by COS-7 cells overexpressing mouse MT1-MMP was inhibited by both E2_C6 and A4_7 showing that these Fc-scFv's are cross-reactive between human and mouse MT1-MMP (Supplementary Figure 2). Importantly, non-transfected COS-7 cells showed no degradation of fluorescent-labeled gelatin. We examined the effect of E2_C6 on cell-free gelatin cleavage using the catalytic domain of MT1-MMP. Although not showing full inhibition of gelatin degradation at the concentrations used, there was a clear inhibitory effect with maximal inhibition of 50 nM MT1-MMP achieved with 100–200 μM E2_C6. The Fc-scFv DES control showed no activity whereas GM6001 showed full inhibition (Supplementary Figure 3).

We used an inducible MT1-MMP overexpressing variant of HT-1080, HTC-75 [27] to evaluate the effect of Fc-scFv's on the level of CD44 shed from the cell surface by MT1-MMP. This cell line displays a low level of endogenous CD44 shedding in part due to MT1-MMP activity, but substantial over-expression is achieved by removal of tetracycline [27, 47]. The inhibition of both endogenous and exogenous MT1-MMP thus demonstrates the cell-surface inhibitory potential of the antibodies. HTC-75 cells cultured in the presence of MT1-MMP-specific Fc-scFv's showed a decreased level of CD44 in the conditioned medium compared to a negative Fc-scFv DES control, whereas CT1746 showed near complete inhibition (Figure 7C). The *IC*_50_ values for the best performing Fc-scFv's A4_7 and E2_C6 were around 100 nM. As a final measure of cell-surface MT1-MMP activity, we examined the effect of antibodies on transwell collagen invasion by HT1080 cells. Fc-scFv A4_7, E2 and C6 showed full inhibition at 500 nM (data not shown). Fc-scFv E2_C6 was tested at different concentrations and, as shown in Figure 7D, displayed near 100% inhibition at 19 nM.

### Anti-MT1-MMP Fc-scFv's inhibit primary tumor growth and metastasis development

**Figure 8:**
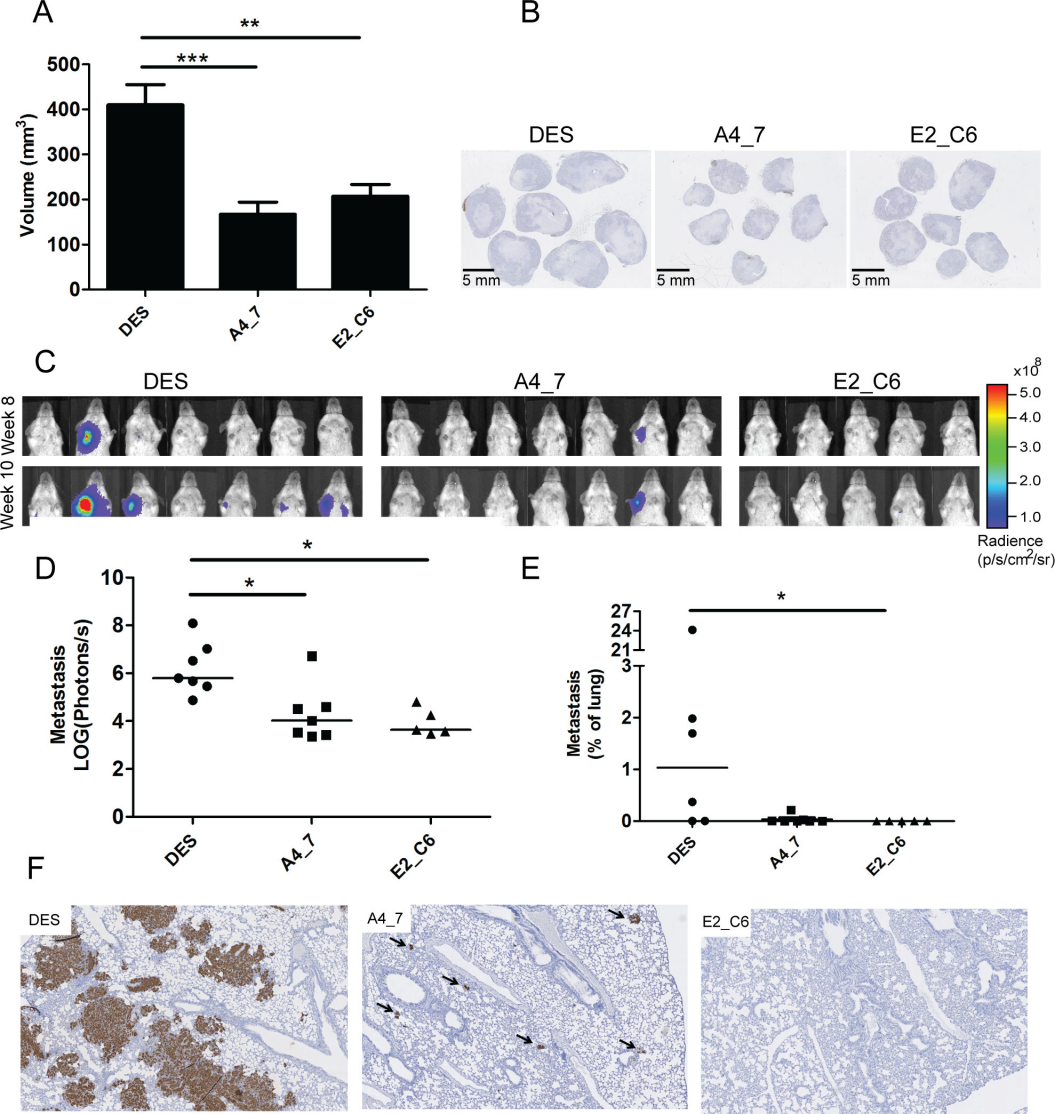
Anti-MT1-MMP A4_7 and E2_C6 Fc-scFv's inhibit primary tumor growth and spontaneous metastasis development in mice MDA-MB-231 LUC2 cells (106) were transplanted into the mammary fat pad of CB-17 SCID mice. A4-7 Fc-ScFv, E2_C6 Fc-ScFv or control DES Fc-ScFv (all at 5 mg/kg) were administered at day four and subsequently three times per week for 10 weeks. (**A**) Calculated tumor volumes after seven weeks were compared between the three treatment groups using ANOVA and Turkeys multi-comparison tests, (**p < 0.01, ***p < 0.001). (**B**) Cross sections of the primary tumors from the three different treatment groups. (**C**) After surgical removal of the primary tumors, the animals were imaged for potential lung and axillary lymph node metastasis development by measuring luciferase activity in the thoracic region using an IVIS Spectrum instrument. (**D**) Endpoint measurements (10 weeks after initiation of treatment) of photon radiance per area were compared between the three treatment groups using the Kruskal-Wallis and Dunn's multi-comparison test (*p < 0.05). (**E**) Lung metastasis development was compared between the three treatment groups by calculating the relative metastasis area immunohistochemically stained for human vimentin of randomly chosen sections of mouse lungs. (**F**) Immunohistochemical stainings of mouse lungs from the three treatment groups showing regions with the most extensive metastasis development. Arrows depict metastatic foci.

Next we investigated the ability of the two selected Fc-scFv's E2_C6 and A4_7 to inhibit primary tumor growth *in vivo*. To select between different relevant tumor cell lines, we analyzed the levels of MT1-MMP using both an ELISA and Western blotting analysis. MDA-MB-231 breast cancer cells, with and without LUC2, contained substantial amounts of MT1-MMP and compared to other cell lines tested, a significantly higher amount of active MT1-MMP without pro-domain (Supplementary Figure 4). Thus, MDA-MB-231 LUC2 breast cancer cells were inoculated into the mammary fat pad of immunodeficient mice. At day four after cancer cell implantation mice were treated with E2_C6 (*n* = 8, 5 mg/kg), A4_7 (*n* = 8, 5 mg/kg) or control DES (*n* = 8, 5 mg/kg) i.p. three times per week for ten weeks. After seven weeks the primary tumors were surgically removed and tumor volume calculated. The size of the primary tumors were significantly reduced by more than two-fold after treatment with E2_C6 (*p* < 0.01) and A4_7 (*p* < 0.001) when compared to treatment with DES (Figure 8A). Cross sections of the primary tumors also demonstrated the difference in tumor growth between the three treatment groups (Figure 8B).

After surgical removal of the primary tumors, the animals were imaged weekly to quantify the development of lung and axillary lymph node metastasis by measuring the luciferase signal from metastatic tumor cells in the thoracic region using an IVIS Spectrum instrument (Figure 8C). Ten weeks after cancer cell transplantation, A4_7 and E2_C6 treated mice had significantly reduced metastasis compared to control DES-treated mice (Figure 8D). Macroscopic histological analysis identified axillary lymph node metastases in 4/7 animals treated with DES, 0/5 of the E2_C6 treated animals and 1/7 of the A4_7 treated animals. A more detailed investigation of the inhibition of lung metastasis was performed using immunohistochemical stainings for human cancer cells of randomly chosen lung sections from each mouse. No metastasis or single human cancer cells were observed in the mice treated with E2_C6, and thus calculation of the relative area of metastases showed that E2_C6 treatment significantly inhibited lung metastasis development compared to DES treatment (Figure 8E). Reduced amounts of lung metastasis (both in numbers and size) was also observed in animals treated with A4_7 compared to those treated with DES (Figure 8F and Supplementary Figure 5), although the difference did not reach statistical significance when comparing the relative area of metastases. To identify the mechanism underlying the difference in size of the primary tumors and the metastasis in mice treated with MT1-MMP Fc-scFv's versus those treated with control Fc-scFv DES, we examined the morphology of primary tumors and metastasis. The primary tumors of the controls were larger, but appeared morphologically similar to the MT1-MMP Fc-scFv-treated tumors, both having a central necrotic area. The ratio between vital tumor tissue and necrotic tissue were similar for the two groups. For both type of tumors, the border between vital tumor tissue and necrotic tissue contained apoptotic cells, as determined by an antibody against cleaved caspase 3 (Supplementary Figure 6). No difference in the number of apoptotic cells was observed between the two groups. The primary tumors and lung metastasis were also stained for collagen using Sirius Red stain. The primary tumors contained very small amounts of collagen surrounding the relatively few blood vessels within the tumors. For the lung metastasis, the MT1-MMP Fc-scFv-treated animals contained few and small metastases and exhibited normal size collagen layers around the bronchia and blood vessels. The animals treated with control Fc-scFv DES exhibited higher numbers and much larger metastases, and several metastases involved blood vessels where the collagen layer was significantly thinned or almost missing (Supplementary Figure 7).

## DISCUSSION

The concept of inhibitors targeting exosites of metzincin metalloproteinases, to gain specificity of action with respect to individual proteases and even specific substrates is a consequence of the close homology of their Zn containing active site clefts [28]. The development of low molecular weight Zn binding inhibitors over some decades has been hampered by this similarity. Although there are currently a number of more specific inhibitors of this type, most have a rather broad range of action with consequent problems for future applications in the treatment of human pathologies, as well as confusion about their precise targets in model studies [29]. Herein, we describe a strategy to identify and refine specific scFv antibodies that bind outside the catalytic cleft of MT1-MMP and effectively prevent its proteolytic functions at the surface of cells.

Most metzincins have domains outside the catalytic domain that may be involved in macromolecular substrate cleavage specificity, as well as functional motifs within the catalytic domain, but outside the active site cleft, termed ‘exosites’. Further, there are data to show that shape changes, or dimer or oligomer formation can also be part of the macromolecular substrate cleavage process. The concept of targeting extra catalytic motifs is reasonable, but major challenges lie in the paucity of structural information on enzyme-substrate interactions outside the catalytic cleft. More detailed work on the collagenolytic MMPs, particularly MMP-1, has shown that the hemopexin domain is essential for collagenolysis, participating in the modulation of the triple helical structure of the substrate to allow access to the catalytic cleft [26]. Interestingly, Remacle and colleagues [30] targeted the hemopexin domain of MT1-MMP to isolate suitable inhibitors from a library of small ligands from the Developmental Therapeutics Program (DTP) National Cancer Institute/NIH collection. Using a site formed by β-strands 1 of blades I to IV of the hemopexin domain of MT1-MMP, distinct from the dimerization interface defined by the crystal structure [31], compound docking simulations were carried out, followed by biochemical and cell-based screening assays to detect potential interacting/inhibitory compounds. They identified a novel compound that interfered with homodimerisation of MT1-MMP but had no inhibitory activity in small fluorescent peptide substrate assays. The compound inhibited tumor cell migration on collagen and effectively repressed tumor growth in an *in vivo* mouse model, confirming that the dimer form of MT1-MMP was necessary for optimal cell surface function [32] Similarly, synthetic peptides mimicking the outermost strand motifs within the hemopexin domain of MT1-MMP defined as essential for homo/hetero dimerisation were shown to specifically inhibit MT1-MMP-enhanced cell migration, although the ability to directly prevent MT1-MMP proteolytic activity was not shown [33]. Furthermore, these peptides interfere with cancer metastasis without affecting primary tumor growth.

Antibodies as inhibitors of metzincin metalloproteinases have also been used to screen for relevant extra catalytic motifs. We have previously developed effective inhibitory antibodies to the disintegrin, cysteine-rich (Dis- Cys) domain of human ADAM17, as well as a cross-domain antibody binding, in addition to the Dis-Cys domain, to catalytic domain exosite(s) [34]. Further, an inhibitory antibody to an exosite motif in murine and human ADAM17 was isolated [35]. Larkin and colleagues developed an antibody to ADAMTS5 that cross-links the active site and the disintegrin domain that was more effective than binding the active site alone [36]. An antibody to the motif Trp116 to Lys214 outside the Zn binding site of MMP-9 was shown to be an effective inhibitor that functioned both *in vitro* and *in vivo* [37]. A monoclonal antibody that recognizes the ‘MT-loop’ structure, an eight residue insertion in the catalytic domain specific for MT–MMPs and distant from the MT1–MMP active site, was developed by Ingvarsen and colleagues and shown to prevent tissue inhibitor of metalloproteinases-2 (TIMP-2) association with MT1–MMP [38] The antibody thus incapacitates the TIMP-2-dependent MMP-2-activating function alone rather than the general enzymatic activity and the pro-migratory function of MT1–MMP [22, 39]. Another antibody to the MT-loop was shown to inhibit HT1080 cell invasiveness by preventing localization of MT1-MMP to the focal adhesion complex without affecting its proteolytic activity [40]. These effects may be compared with a monoclonal antibody to a peptide describing the MT-loop originally prepared by the Arroyo group. A recent crystal structure of its interactions with MT1 MMP has shown that conformational swiveling of the surface MT-loop is required for effective binding and consequent inhibition of MT1-MMP activity on the cell membrane. This inhibition mechanism appears to effectively control active MT1-MMP in endothelial cells and at the leading edge of migratory cancer cells [23].

Phage display libraries are powerful tools to identify a repertoire of different antibody binding motifs in target antigens. The consequent antibodies are amenable to improvements by both engineering or maturation techniques, as well as subsequent large-scale production as recombinant proteins [41]. In the case of MT1-MMP, an inhibitory antibody fragment (DX-2400; Ki = 0.6 nM for human MT1-MMP; IC50 ∼ 1–5 nM) was developed using a human Fab library and the MT1-MMP catalytic domain as antigen [20]. This antibody can be inferred to interact with the active site cleft since it inhibited small fluorescent peptide hydrolysis. It was found to inhibit MDA-MB-231 primary tumor growth and metastasis in xenograft mouse models, although problems with *in vivo* stability have been reported [18].

In the quest for suitable therapeutic entities, we set out to examine whether additional inhibitory specificity might be gained by targeting extra catalytic motifs of MT1-MMP in screens of highly versatile antibody fragment libraries displayed on bacteriophages. Previously, we described a recombinant human scFv antibody against the hemopexin domain of MT1-MMP derived from the screening of a V-gene library [21]. The lead scFv modulated MT1-MMP interactions with collagen, and was weakly inhibitory in an isolated collagen fibril assay. In cell-based studies, it did not inhibit MT1-MMP-mediated pro-MMP-2 activation but effectively prevented cell degradation of collagen or gelatin films and cell membrane CD44 shedding. Further, it was found to be an effective inhibitor of the invasive capacity of cancer cells and of angiogenesis in model systems [12, 21], but no *in vivo* experiments were performed.

In the study described herein, we used a more powerful phage display library reported to yield antibodies with high affinity and diversity [24, 42, 43], screening against the full ectodomain of pro-MT1–MMP. With the propeptide blocking the active site cleft, we searched for antibodies binding outside the conserved active site. Our approach focused on identifying antibody fragments that were effective inhibitors of cell-surface MT1-MMP. Instead of targeting a specific domain of MT1-MMP, the phage library was subjected to the ectodomain and the screening for inhibitory clones was directed towards MT1- MMP on the surface of living cells as early as possible with the specific aim of isolating scFv antibodies that effectively inhibit a range of cell-associated MT1- MMP proteolytic events. It has been reported that compounds to cell-membrane proteases displaying low nanomolar inhibition in biochemical assays are much less potent inhibitors of their cell-membrane-bound target [44], stressing the importance of early screening towards the true cell-membrane-bound target molecule. Biochemical assays with small and macromolecular substrates for MT1- MMP showed that the lead Fc- scFv E3 did not interfere directly with small substrate or inhibitor binding to the active site cleft. Instead, lead Fc-scFv's interfered with the binding of macromolecular substrates and inhibitors to the catalytic domain. This was exemplified by Fc- scFv inhibition of catalytic domain-TIMP-2 interactions and gelatin degradation by the MT1- MMP catalytic domain [32], showing that Fc-scFv's act as orthosteric antagonists competing with the enzyme interactions to macromolecular substrates.

However, the lack of binding competition between selected lead Fc-scFv's and one MT1-MMP ligand used in this study, a triple helical collagen peptide, is more difficult to interpret. This peptide binds both the active site cleft and the hemopexin domain of MMP1 (DB133) [25, 26] and is also known to bind MT1-MMP (B. Basu, M. Rapti and G. Murphy; unpublished). Thus, the collagen peptide likely spans a larger region of the MT1-MMP surface as on MMP-1, but is not competed away by the presence of Fc-scFv E3.

Having identified several lead clones, we proceeded to utilise a maturation process to enhance their affinity. Since highly diverse CDR3 loops have been reported as the key determinant of specificity in antigen recognition [45], we carried out affinity selections using our most potent first generation scFv clones E3 and G1. New VH and VL libraries were constructed via randomizing blocks of five to six amino acid residues in the CDR3 loops [35, 46]. Using affinity selections with steadily decreasing antigen levels in addition to off-rate selection with a large excess of non-biotinylated antigen, antibodies with changes in the CDR3 loops that decreased the dissociation rate and increased overall binding strength were favoured. Since first-generation antibody fragments were selected for their ability to inhibit cell-surface MT1- MMP activity, screening for 2nd generation higher affinity antibody fragments was carried out such that the binding epitope and inhibition mechanism was preserved. Thus, a homogenous time-resolved fluorescence assay using competition with parent Fc-scFv E3 as measure was used in combination with the ability to compete with TIMP- 2 for MT1-MMP binding. The finally selected mature clones were assessed for their activity in cell-associated MT1-MMP assays and found to be active against pro-MMP-2 activation, gelatinolysis, collagenolysis (HT1080 cell migration) and CD44 shedding [47]. The inhibitory activity towards cell-surface pro-MMP-2 activation might be a combined effect of sterically interfering with MT1- MMP interactions with both TIMP-2, functioning as a pro-MMP-2 activation ‘receptor’, and the pro- MMP- 2 substrate. In addition to these cell-surface effects, E2_C6 inhibited cell-free gelatin degradation catalyzed by catalytic domain MT1- MMP. We conclude that the motif(s) targeted by our Fc-scFv's therefore represent a common interaction site(s) for a number of different macromolecular substrates and that the site(s) are localized in the catalytic domain outside the active site cleft. A hypothesis on the epitope localization can be inferred based on both of the following two parameters; residues that differ between MT1-MMP and MT2-MMP and residues within the proximity of the MT1-MMP-TIMP-2 interaction site. By protein sequence alignment of MT1- MMP and MT2-MMP and from inspection of the crystal structure of the complex between TIMP-2 and MT1-MMP [Protein Data Bank accession 1BQQ], the following amino acids were identified as potential epitope residues: I114L, Q115T, L117R, A165E, Y166D, E169L, G170R, H171R, E172Q, Q174E, E183S, T190S, E195T, N208G, I209L, S217A, A218D, N229H, D252N, S254N, M264K and await future structural validation.

The two most active Fc-scFv antibodies were tested in an MDA-MB-231 tumor model using CB-17 SCID mice and found to slow tumor growth significantly and to completely abrogate the development of metastases. This is similar to the work of Zarrabi *et. al.* [33], wherein peptides binding the MT1-MMP hemopexin domain prevented MDA-MB-231 metastases, but had little effect on primary tumor growth.

Thus, targeting extra-catalytic domain motifs using an antibody library represents a novel approach for the inhibition of MT1-MMP-mediated cellular proteolysis. Given the role of this enzyme in cell invasive processes associated with cancer, rheumatoid arthritis, atherosclerosis and diabetes, future rigorous testing of the antibodies in a wide range of pathological models should be investigated. This will allow further evaluation of the importance of MT1-MMP mediation in the migration of different cell types driving various disease processes and determine the shape of novel therapeutic strategies.

## MATERIALS AND METHODS

### Proteins and reagents

Variants of MT1-MMP; pro-MT1-MMP E240A (full ectodomain of MT1-MMP containing the pro-domain, residues 21–541 and with the E240A mutation); MT1-MMP wild-type (wt) without pro-domain (residues 112–541, ectodomain); wt MT1-MMP catalytic domain (residues 112–285); and wt MT1-MMP hemopexin domain (residues 316–508) were expressed in *Escherichia coli* BL21 (DE3) pLysS (Invitrogen), refolded from inclusion bodies and purified using ion exchange chromatography (HiTrapQ FF, GE healthcare) followed by gel filtration (for pro-MT1-MMP E240A). Pro-MT1-MMP E240A was analyzed by N-terminal Edman sequencing to confirm presence of pro-domain. Pro-MT1-MMP E240A was biotinylated using biotin-xx-microscale protein labeling kit (B30010, Invitrogen) following manufactures recommendations. The degree of labeling was estimated to be 1–2 molecules of biotin/molecule of enzyme (FluoReporter Biotin Quantitation Assay Kit, Invitrogen). MT2-MMP, MT3-MMP, MT5-MMP, MMP- 1, MMP-2, MMP-3, MMP-7, MMP-9, MMP- 10, MMP-13, MMP- 19, MMP-26 were available from R & D and ADAM17 produced in-house [34]. TIMP-2 and TIMP-3 with C-terminal His-tag was expressed in *E. coli*, folded *in vitro* and purified [48]. The triple helical collagen II peptide DB133 was a kind gift of Richard Farndale, Biochemistry, Univ. Cambridge, UK [25]. Unless stated otherwise, all chemicals were purchased from Sigma-Aldrich. The hydroxamate inhibitor GM6001 was from EMD Millipore (Temecula, CA, USA) and CT1746 was a gift from UCB/Celltech, Slough UK.

### Phage display selections

A naïve human scFv phage-display library [24] was used for selection of MT1-MMP binding antibodies. Initially, the library was pre-incubated with streptavidin-beads (Dynal beads, Invitrogen) to remove phages recognizing the beads before being exposed to 100 nM of biotinylated pro-MT1-MMP E240A. Streptavidin-beads were used to retrieve phages displaying antibody fragments binding to biotinylated pro-MT1-MMP E240A. Following two rounds of solution-phase selection, the eluted polyclonal scFv population was cloned into the pSANG10–3F expression vector for *Escherichia coli* BL21 (DE3) bacterial expression of scFv [49]. Individual clones (total of 960) were isolated and expressed in 96-well format and culture supernatants directly tested for specific MT1-MMP binding by ELISA screening (See M & M on ELISA below). The 192 clones showing highest specific ELISA signal were sequenced and further evaluated for inhibitory activity.

### V_H_ and V_L_ CDR3 randomized library generation and phage selection

For affinity maturation of lead candidate antibody fragments, E3 and G1, the V_H_ and V_L_ CDR3 loops were randomized in blocks of 5–6 amino acid residues using kunkel mutagenesis as previously described [35, 46, 50]. In brief, uracil-containing ssDNA (dU-ssDNA) encoding the parental E3 or G1 with stop codons was purified from M13 phage using the QIAprep spin M13 kit (Qiagen). Primers encoding the desired randomized region were used to generate covalently closed circular double-stranded heteroduplex DNA (ccc-ds(U)DNA) consisting of wt and mutated DNA. The ccc-ds(U)DNA were transformed into DH5α cells leading to degradation of the wt DNA strand. Subsequent sequencing showed an average of approximately 70% of clones being mutated in the desired region. To increase the fraction of mutated sequences, a method incorporating rolling cycle amplification favouring the mutated DNA was utilized. The final library DNA was PCR purified (Fermentas GeneJet PCR purification kit) and transformed into freshly prepared electrocompetent TG1 cells that were spread on Bioassay plates. The size of each library was between 2 × 10^9^ and 8 × 10^9^ covering the theoretical diversity of approximately 1 × 10^9^ DNA molecules. Library DNA was sequence confirmed showing that nearly 100% of clones were mutated. For phage display selections, dsDNA mutant phagemid libraries were rescued directly from the TG1 cells.

During phage display selections, V_H_ and V_L_ libraries were kept separate. Titrated concentrations (from 10 to 0.05 nM) of biotinylated human pro-MT1-MMP E240A were exposed to the CDR3 randomized V_H_-/V_L_-libraries. Three rounds of affinity solution-phase selection were carried out before 1 round of off-rate selection on which binding of antibody phages to biotinylated pro-MT1-MMP E240A was allowed to reach equilibrium (2 hour incubation) before addition of a 100-fold excess of non-biotinylated human pro-MT1-MMP E240A. Binding reactions were then allowed to proceed for 1, 5 and 20 hours. Fast-dissociating antibody fragments would most likely re-bind non-biotinylated MT1-MMP and therefore not be selected when using streptavidin-beads. Following affinity and off-rate selections, eluted polyclonal scFv populations were cloned into pSANG10- 3F and transformed into *Escherichia coli* BL21(DE3) for expression along with parental scFv-E3 and scFv-G1. A total of 1152 new scFv clones were individually selected and expressed in *E*. *coli*, and the culture supernatants used directly for screening using quenched-fluorescent peptide cleavage assay and homogenous time-resolved fluorescence (HTRF) assay (See M & M below).

### Antibody formatting and expression

Antibodies were used in either the scFv or the Fc-scFv format, as indicated. For scFv-fragments, expression was carried out using the pSANG10-3F vector and *Escherichia coli* BL21(DE3) cells [49], and the scFv-fragments were used from the culture supernatant directly or from the periplasmic extract with purification by Ni-NTA (Generon). For Fc-scFv antibodies, the heavy and light chains were cloned into pBioCAM5 and transfected into HEK-293E suspension cells for mammalian cell expression, as described [49]. Antibody fragments were purified from conditioned media using protein A Sepharose CL-4B (Invitrogen) or Ni-NTA agarose (generon) and AKTA FPLC affinity chromatography (GE Healthcare). Fc-scFv antibodies were dialysed into 50 mM Tris, 150 mM NaCl, 10 mM CaCl_2_, pH 7.4 (TNC) and filter-sterilized.

### Collagen type I cleavage assay

For screening of antibody fragments towards inhibition of MT1-MMP catalyzed collagen cleavage, scFvs were individually expressed using *Escherichia coli* BL21 (DE3) in 10 mL auto-induction media. Periplasmic fractions were affinity-purified by Ni-NTA Agarose (Qiagen) [51] followed by buffer-exchange into 50 mM Tris, 150 mM NaCl, 10 mM CaCl_2_, pH 7.4 with 0.05% Brij35 (TNC-Brij) using 96-well Zeba spin filter plates (Thermo Scientific). Collagen type I fibrils were formed by incubation at 35°C, 2 hours and scFv antibodies were pre-incubated with 20 nM active MT1-MMP (residues 112–541) for 30 minutes, room temperature (RT). Fibrillar collagen was mixed with pre-incubated MT1-MMP/scFv and allowed to react for 16 hours at RT before being analysed by SDS-PAGE. From an initial screening of 192 clones, those showing inhibition of collagen cleavage were expressed at a 50 ml scale, purified as above, and re-tested using a fixed concentration of 1.5 μM scFv. As controls, anti-desmin antibody (Fc-scFv DES) (1.5 μM), CT1746 (10 μM) and no MT1-MMP was used.

### ELISA for initial scFv antibody screening, collagen-MT1-MMP binding and detection of MT1-MMP levels in cell lysates

For initial ELISA screening of monoclonal antibody fragments binding to MT1-MMP, Streptavidin in PBS was added to Nunc MaxiSorp^™^ plates (Nunc) for overnight incubation at 4°C. Plates were blocked with 3% BSA in TNC for 1 hr at RT and washed 3 times in TNC with 0.1% Tween-20 (TNC-T), then 3 times in TNC before biotinylated pro-MT1-MMP E240A (3 μg/ml) in TNC with 1.5% BSA was added. Culture supernatants from bacterial expression containing scFv antibodies were diluted 2-fold in 2xTNC with 3% BSA and applied directly to the ELISA plates. Following 1 hour incubation and wash as above, binding of antibodies was detected with anti-FLAG europium in TNC with 1.5% BSA using time-resolved fluorescence spectroscopy.

For detecting MT1-MMP binding to collagen in the presence of Fc-scFv's using ELISA, collagen type I (13 mg/ml) and collagen peptide DB133 (5 mg/ml) [26] were diluted 1000-fold in TNC (collagen type I) and in 10 mM acetic acid (collagen peptide) and applied to Nunc MaxiSorp^™^ plates (Nunc) for overnight incubation at RT. Plates were blocked with 3% BSA in TNC-T and washed in TNC-T before 25 nM biotinylated pro-MT1-MMP E240A in the absence or presence of E3 Fc-scFv (0–500 nM) or DES Fc-scFv (500 nM) was added and allowed to bind collagen for 30 minutesat RT. Plates were washed 3 times in TNC-T, and binding of MT1-MMP to collagen was detected using HRP-streptavidin diluted in TNC-T and 3, 3′, 5, 5′-Tetramethylbenzidine (TMB) substrate with absorbance measured at 450 nm.

To detect levels of MT1-MMP in cell lysates from human cancer cell lines, polyclonal rabbit anti-MT1-MMP (epitomics 2010–1) was diluted 200-fold in 100 mM Na_2_CO_3_, pH 9.6 and applied to wells of Nunc MaxiSorp^™^ plates (Nunc). To avoid MT1-MMP activity in ELISA, 10 μM CT1746 was applied to all subsequent steps. Plates were blocked in TNC-T with 3% BSA and cell extracts (extraction buffer: 0.1 M tris, pH 8.1 m 10 mM EDTA, 0.5% triton X-100) was diluted 10-fold into TNC-T before being applied to plates. After 3 times wash in TNC-T, MT1-MMP was detected with polyclonal sheep anti-MT1-MMP N175/6 and donkey-anti-sheep-HRP using TMB substrate and absorbance measured at 450 nm.

### Quenched-fluorescent peptide cleavage assay with and without TIMP

Initial screening of scFv antibodies in quenched-fluorescent peptide cleavage assays was carried out using scFv antibodies purified from 10 ml *Escherichia coli* BL21 (DE3) cultures as described above. For affinity-matured scFv antibody screening, culture supernatants were used directly without purification but with a 200-fold final dilution into the assay buffer TNC-brij. Assays were performed with 0.5–2 nM MT1-MMP pre-incubated alone or in the presence of scFv or purified Fc-scFv antibodies, as indicated. Cleavage of the fluorogenic peptide substrate Mca-KPLGL-Dpa-AR-NH2 (1 μM) (SMO-3670-PI, Peptides International) in the presence or absence of 15 nM TIMP-2 was followed by excitation at 320 nm and emission recorded at 405 nm in a Tecan Infinite-200, at 37°C.

To examine specificity of inhibitors, 0.5–20 nM of each proteinase (MT1-MMP catalytic domain or ectodomain, MT2-MMP, MT3-MMP, MT5-MMP catalytic domain, MMP-1, MMP-2, MMP-3, MMP-7, MMP-9, MMP-10, MMP-13, MMP-19, MMP-26 and ADAM17) were pre-incubated with a final of 200 nM of Fc-scFv antibodies for 30 minutes at RT. TIMP-2 or TIMP-3 with 1 μM Mca-KPLGL-Dpa-AR-NH2 substrate were then added and peptide cleavage performed as above.

### Homogeneous time-resolved fluorescence (HTRF) assay

Initially, parent antibody Fc-scFv E3 was labeled with d2-acceptor per manufacturer's recommendations (d2-labeling kit, Cisbio) to obtain 2–3 d2 molecules/molecule Fc-scFv E3. A HTRF standard curve of d2-Fc-scFv E3 binding to biotinylated pro-MT1-MMP E240A (0.01–200 nM) was prepared using streptavidin europium cryptate donor (Cisbio) and the HTRF signal was read using excitation at 340 nm and emission at 620 (donor) and 665 (acceptor). HTRF competition assays were carried out using a mix of 0.5 nM biotinylated pro-MT1-MMP E240A and streptavidin europium cryptate pre-incubated with 8-fold diluted *Escherichia coli* BL21 (DE3) culture supernatant containing mutated scFv antibodies. Following 30 minutes of incubation at RT, 3 nM d2-labelled parent Fc-scFv E3 was added and HTRF signal read as above. Controls were non-labeled E3 parent and DES.

### Pro-MT1-MMP activation assay

Pro-MT1-MMP E240A (2 μM) was preincubated with Fc-scFv's (3 μM) for 30 minutes, RT, in 100 mM HEPES, 10 mM CaCl_2_, 0.5% triton X-100, before 1 unit of furin (NEB) was added. After 3 hours at 30°C, products were analyzed by non-reducing SDS-PAGE.

### Surface plasmon resonance analysis

Surface plasmon resonance (SPR) analyses were performed on a BIACORE T100 instrument using CM5 sensor chips and a buffer of 30 mM Hepes, 135 mM NaCl, 10 mM CaCl_2_ with 0.1% BSA and 0.05% Tween 20 (running buffer). Affinities of the Fc-scFv's for wt or various forms of MT1-MMP were determined by pre-capturing the Fc-scFv's on anti-human Fc (GE Healthcare), immobilized to 10000 RU, by injecting 5 μl of 50 nM Fc-scFv antibody. The protease (0.1–200 nM) was thereafter injected for 120 seconds followed by a dissociation of 600 seconds and with a flow rate of 30 μl/min. Binding of the Fc-scFv's to preformed complexes between MT1-MMP wt catalytic domain (50 nM) and TIMP-2 (1 μM) or CT1746 (10 μM) was carried out in the same manner as above. To confirm kinetics of E2_C6 showing slow dissociation, a single-cycle set-up was utilized in which increasing concentrations of MT1-MMP were injected over captured E2_C6 as above, but with only one dissociation phase of 7200 seconds. To ensure a stable chip surface between cycles, one concentration of protease was injected at least twice for each antibody or protease variant. The *k*_on_, *k*_off_ and *K*_D_ values were determined by global fitting to a 1:1 binding model with the BIACORE evaluation program.

### Gelatin and collagen film degradation assay

Cell-based MT1-MMP-dependent gelatin film degradation assay was carried out as described previously [52]. Briefly, HT1080 cells were cultured on coverslips coated with AlexaFluor488 -labelled gelatin (F-gelatin) in the presence or absence of Fc-scFv's or GM6001 (10 μM). After 18 hours of incubation, cells were fixed with 3% paraformaldehyde in PBS and stained with Alexa Fluor 568 phalloidin (Invitrogen), and DAPI (4′, 6-diamidino-2-phenylindole) (Invitrogen). Coverslips were imaged with fluorescent microscopes using objective lens of 10×. Areas of fluorescent-labeled gelatin degradation were observed as black zones on green background. Areas of degradation were analyzed using Volocity software (Perkin-Elmer).

For cell-free gelatin degradation, gelatin was incubated with or without 50 nM catalytic domain MT1-MMP and in the presence of E2-C6 (0–200 nM), DES (200 nM) or GM6001 (10 μm). After incubation, the reaction products was analysed by SDS-PAGE. For quantifying gelatin degradation, the gelatin fragment of 35 kDa was used and analyzed by densitometry.

The collagen-film degradation assay was carried out as described previously [52]. Briefly, pepsin-extracted type I collagen (PureCol, advanced Biomatrix) was neutralized and then thinly coated onto 12-well culture plates. After incubation at 37°C for three hours, HT1080 cells were seeded on the collagen film in the presence or absence of Fc-scFv's or GM6001 (10 μM). After 3 days incubation, cells were removed by trypsin to reveal collagen film. Remaining collagen were fixed with paraformaldehyde and stained with Coomassie Brilliant Blue. Areas of collagen degradation were observed as white zones on blue background under widefield microscopy.

### Cellular Pro-MMP-2 activation assay

HT1080 cells in 12-well plates were treated with 100 μg/ml type I collagen (PureCol, advanced Biomatrix) in serum-free media in the presence or absence of Fc-scFv's or GM6001 (10 μM). After 3 days, conditioned media were analyzed for pro-MMP-2 activation by zymography, and cells lysates were analyzed for MT1-MMP and actin by Western Blotting. Zymography was performed using Novex 10% gelatin zymogram gels (Invitrogen) according to manufacturers’ instructions. Levels of pro-MMP-2 and active MMP-2 were quantified using Image J.

### CD44 shedding cell assay

Tet-Off HTC-75 cells, which overexpress MT1-MMP [47], were used for CD44 shedding assays. These cells were seeded in 12-well cell culture plates (Corning) and grown 24 hours without doxycycline to upregulate MT1-MMP expression. Culture media were then switched to serum-free DMEM in the presence or absence of Fc-scFv's or GM6001 (10 μM). After 15 hours, shed CD44 in the conditioned media was analyzed by ELISA (CD44 instant ELISA, eBiosciences) according to manufacturer's instructions.

### Cell invasion assay

The collagen invasion assay was performed as described previously with modifications [40]. Briefly, the upper side of 8 μm pore membranes of transwell chambers (Fisher Scientific) were coated with neutralized acid-extracted type I collagen (cell matrix type I-A, Nitta gelatin). In the lower chamber, media containing 10% FBS with or without Fc-scFv's or GM6001 (10 μM) were added. 3 × 10^4^ HT1080 cells in serum-free medium were seeded on the collagen matrix of the upper chamber with or without Fc-scFv's or GM6001 (10 μM). After incubation for 24 hours at 37°C, invaded cells appearing on the other side of the membrane were counted by staining cells with DAPI and analysing images with Volocity software.

### Western blot analysis

Analysis of cell lysates for the presence of MT1-MMP by Western blotting was carried out using a sheep anti-MT1-MMP polyclonal antibody (N175/6) [53]. As a loading control, actin was detected by rabbit anti-actin polyclonal antibody (Abcam).

### Cell lines and culture

The MDA-MB-231 LUC2 [54] cell line was maintained in DMEM supplemented with 10% FBS, 5% penicillin/streptomycin. Cultures were grown in a humidified atmosphere of 5% CO_2_ at 37°C.

### *In vivo* studies of anti-MT1-MMP treatment

Subconfluent MDA-MB-231 cells were washed in PBS and harvested using trypsin. Cells (1 × 10^6^) were resuspended in a 1:1 mixture of extracellular matrix from the Engelbreth-Holm-Swarm sarcoma and DMEM before orthotopic transplantion into the mammary fat pad of anesthetized eight week-old female CB-17 SCID mice (Taconic, Ry, Denmark). E2_C6 Fc-scFv, A4_7 Fc-scFv and control DES Fc-scFv (all at 5 mg/kg) were injected *i.p*. four days after the transplantation (*n* = 8) and subsequently injected three times per week for ten weeks. Primary tumors were removed from anesthetized mice using blunt instrument dissection. Primary tumor sizes were calculated using V = (W^2^ × L)/2. All animal experiments were approved by The Experimental Animal Committee, The Danish Ministry of Justice and performed at the animal facility core at University of Southern Denmark. The mice were housed under specific pathogen-free conditions with *ad libitum* food and drinking water. The mice were euthanized if they showed any adverse signs or symptoms of disease, including weight loss, paralysis or general discomfort.

### *In vivo* imaging

Relative quantification of metastasis development was performed weekly using whole-body bioluminescent imaging (IVIS-spectrum, Caliper Life Science). Mice were injected with D-luciferin (150 mg/kg body weight) and then anaesthetized with isoflurane gas. Images were acquired starting 10 min after luciferin injection. Regions of interest (ROI) were drawn to encircle the thoracic region to quantify lung and axillary lymph node metastases. The photon emission transmitted from the ROIs was quantified in photons/s/cm^2^/sr using Caliper Life Science Living image (version 4.2).

### Immunohistochemistry

Staining procedures were performed as previously described [55]. For human vimentin (VimentinV9, M0725, Dako, 1:1000) and cleaved caspase-3 staining (ASP175, Cell Signaling Tech, 1:400), heat-induced epitope retrieval was performed by microwave boiling for 15 min in TE (Tris/EDTA, pH 9) buffer (Dako). Collagen staining was performed using Sirius Red stain and analyzed by polarized light microscopy.

### Statistics

The statistical significance of bioluminescence measurements of tumor growth or metastasis in different groups was calculated using one-way ANOVA or Kruskal-Wallis tests, when appropriate. To analyze the specific sample pairs, Turkeys or Dunn's multi-comparison test were used. *p*-values greater than 0.05 were considered not significant.

## SUPPLEMENTARY MATERIALS FIGURES



## Abbreviations

MMP: matrix metalloproteinase
MT-MMP: membrane-type MMP
scFv: single chain variable fragment
ECM: extracellular matrix
DMEM: Dulbecco's modified Eagle's medium
FCS: foetal calf serum
TIMP- 2: tissue inhibitor of metalloproteinases-2
RT: room temperature
TNC: 50 mM Tris 150 mM NaCl 10 mM CaCl2 pH 7.4
wt: wild type
