# Supplementary material for: Development of a specific affinity-matured exosite inhibitor to MT1-MMP that efficiently inhibits tumor cell invasion in vitro and metastasis in vivo
Source: Oncotarget. 2016 Feb 27;7(13):16773–92. doi: 10.18632/oncotarget.7780 (PMC4941350; doi:10.18632/oncotarget.7780)
Supplement: Supplementary file 1 [file oncotarget-07-16773-s001.pdf]

Development of a specific affinity-matured exosite inhibitor to MT1-MMP that efficiently inhibits tumor cell invasion *in vitro* and metastasis *in vivo*

Supplementary Materials

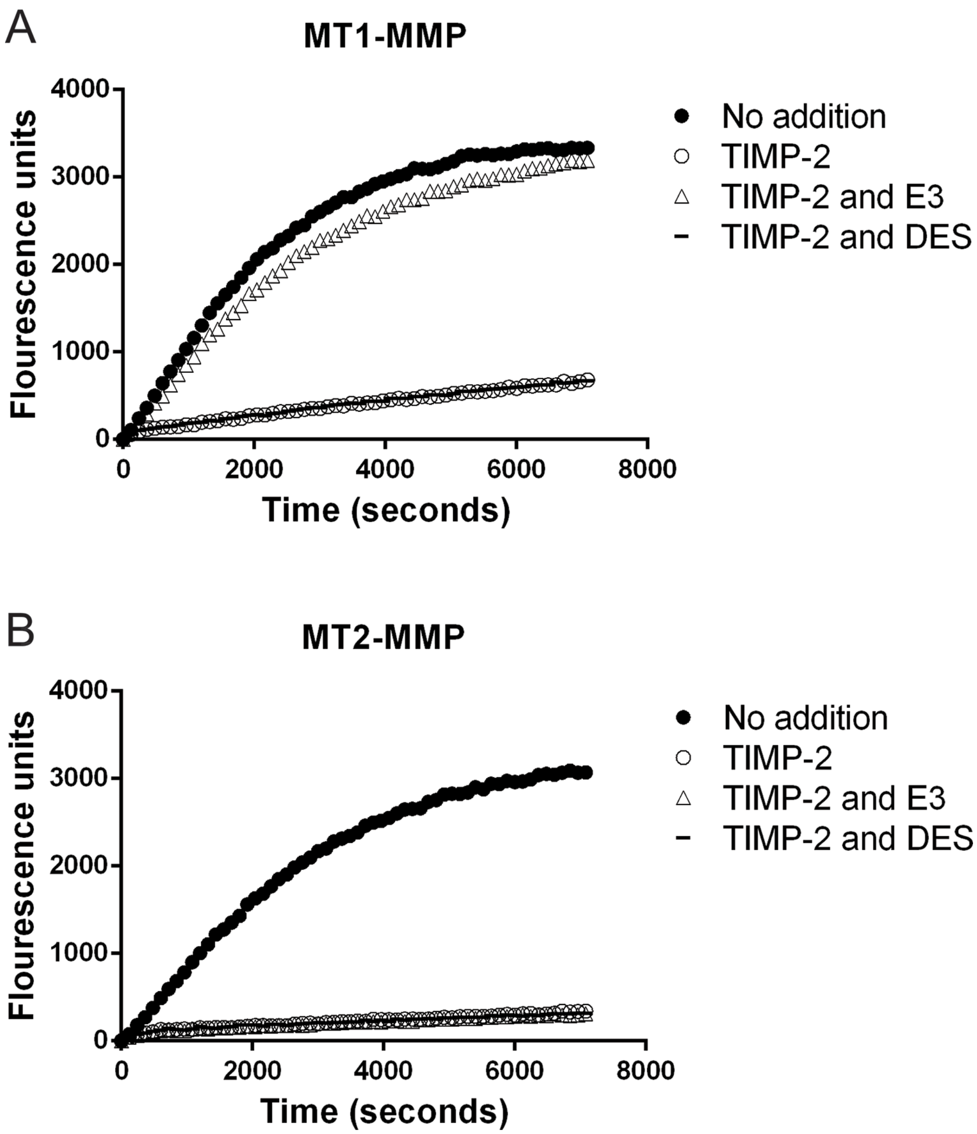

Supplementary Figure S1: Specificity of lead Fc-scFv E3 for MT1-MMP. Cleavage of small fluorogenic peptide by ectodomain MT1-MMP (**A**) or MT2-MMP (**B**) was inhibited by TIMP-2. E3 Fc-scFv outcompeted TIMP-2 inhibition of MT1-MMP but not of MT2-MMP whereas DES control had no effect.

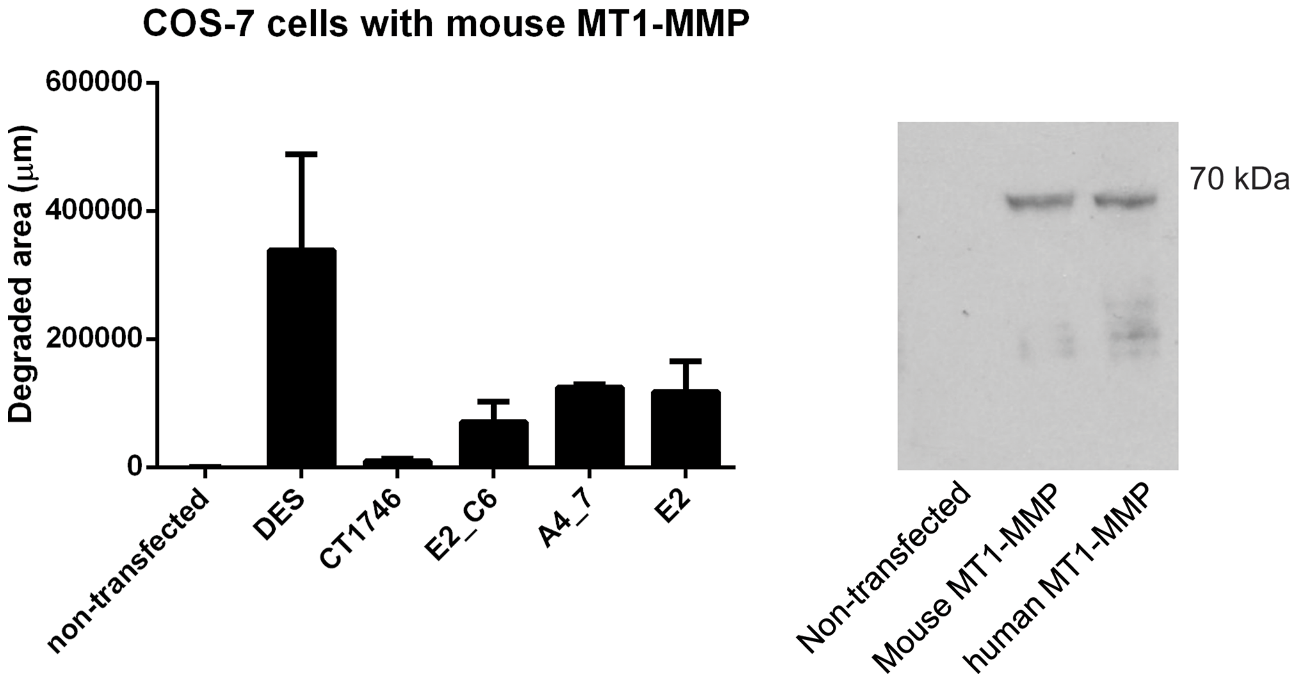

Supplementary Figure S2: MT1-MMP Fc-scFv's inhibit mouse MT1-MMP activity. COS-7 cells were transfected with mouse MT1-MMP and cultured on fluorescent-labeled gelatin in the absence or presence of MT1-MMP Fc-scFv's (500 nM), DES control (500 nM) or CT1746 (10 μM); non-transfected COS-7 cells are also shown. Cells were fixed after 18 hours and imaged. Quantification of gelatin degradation was done by densitometric analysis of inverted images. Western Blotting analysis was utilized to confirm expression of mouse and human MT1-MMP in COS-7 cell using sheep anti-MT1-MMP polyclonal antibody N175/6.

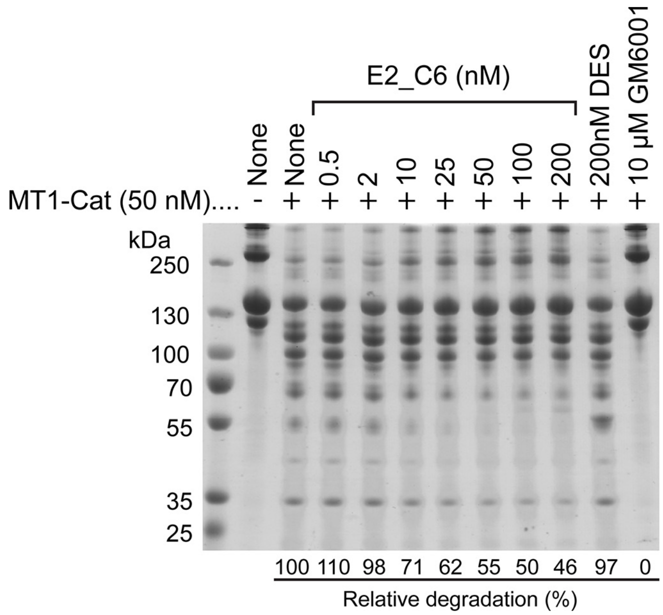

Supplementary Figure S3: Gelatin degradation analysed by SDS-PAGE. Gelatin was incubated with or without 50 nM catalytic domain MT1-MMP and in the presence of anti MT1-MMP Fc-scFv E2-C6 (0–200 nM), control Fc-scFv DES (200 nM) or GM6001 (10 μm). The gelatin degradation was visualized by SDS-PAGE. For quantification of gelatin degradation, the gelatin fragment of 35 kDa was used and analyzed by densitometry.

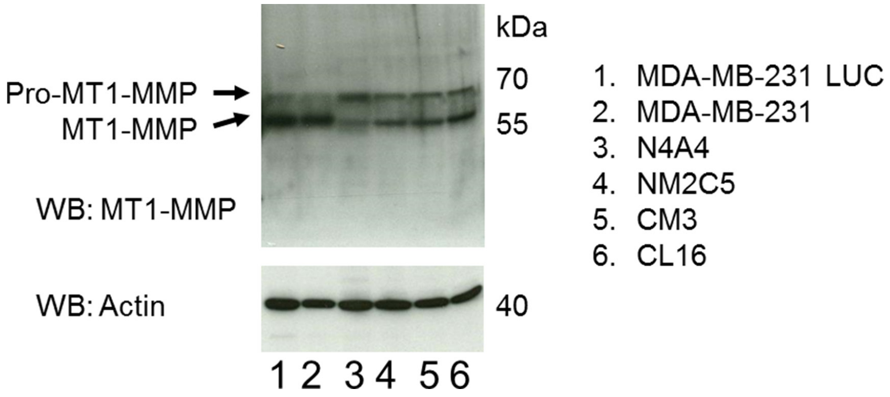

Supplementary Figure S4: Analysis of MT1-MMP in cell line extracts. Levels and type of MT1-MMP in cell line extracts of MDA-MB-231 LUC, MDA-MB-231, N4A4, NM2C5, CM3 and CL16 were assessed using Western blotting analysis with anti-MT1-MMP polyclonal antibody N175/6. As a loading control, actin was detected by rabbit anti-actin polyclonal antibody.

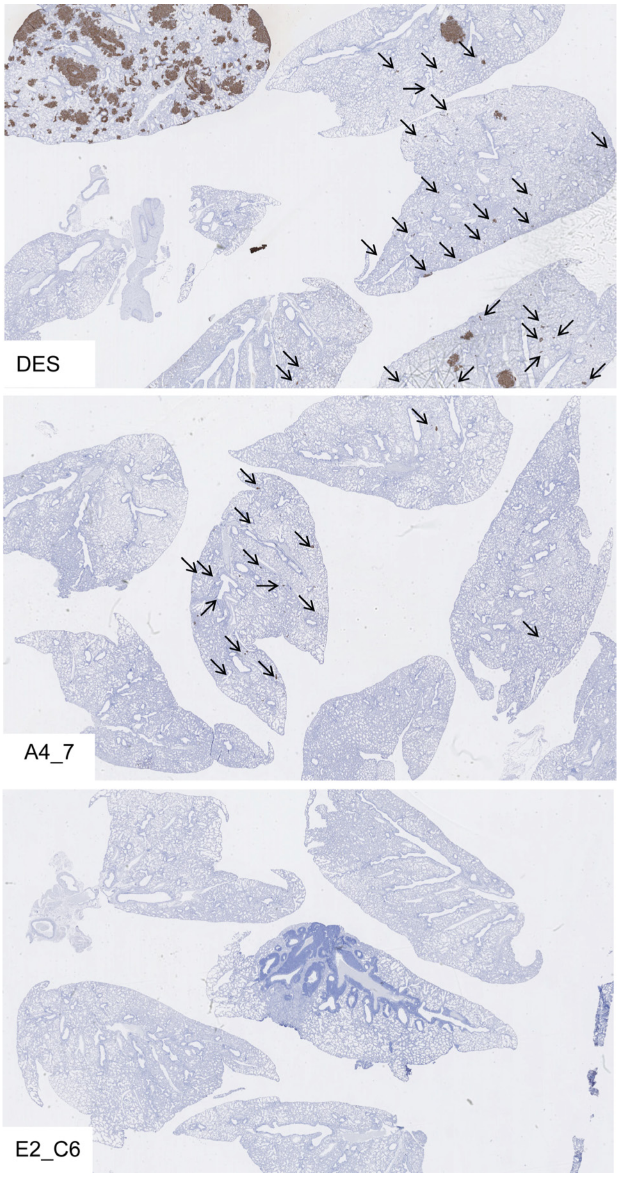

Supplementary Figure S5: Anti-MT1-MMP A4_7 and E2_C6 Fc-scFv inhibit lung metastasis development. Immunohistochemical staining for human vimentin of a lung from each mouse in the three different treatment groups. Arrows depict small metastatic foci.

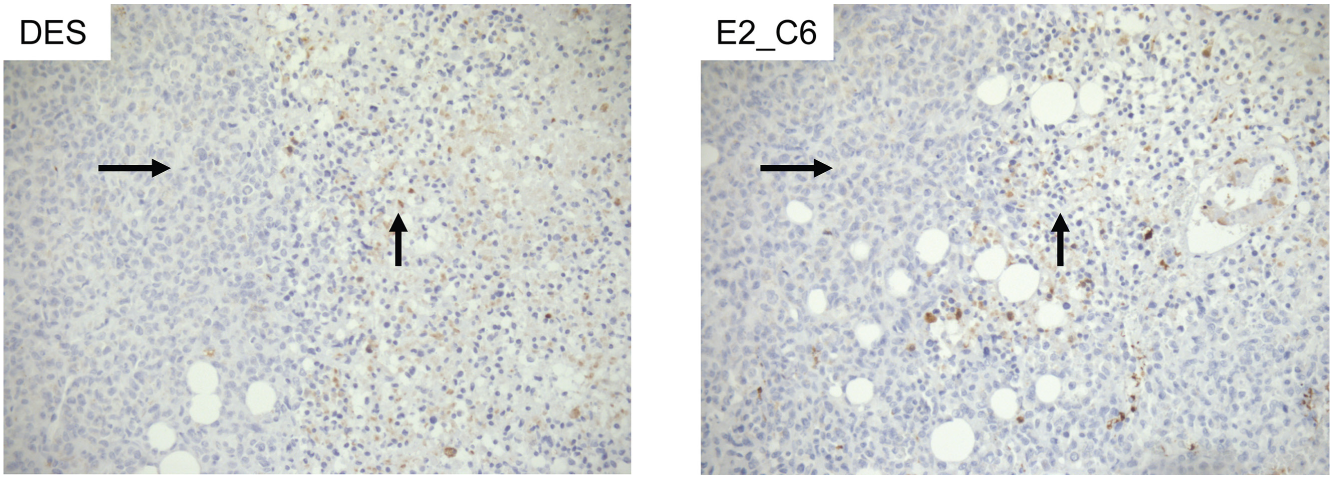

Supplementary Figure S6: Apoptotic cells in the primary tumors of Fc-scFv-treated mice. Primary tumors were stained with anti-cleaved caspase-3 antibody showing the apoptotic cells at the border between the vital tumor tissue (horizontal arrow) and the necrotic tissue (vertical arrow).

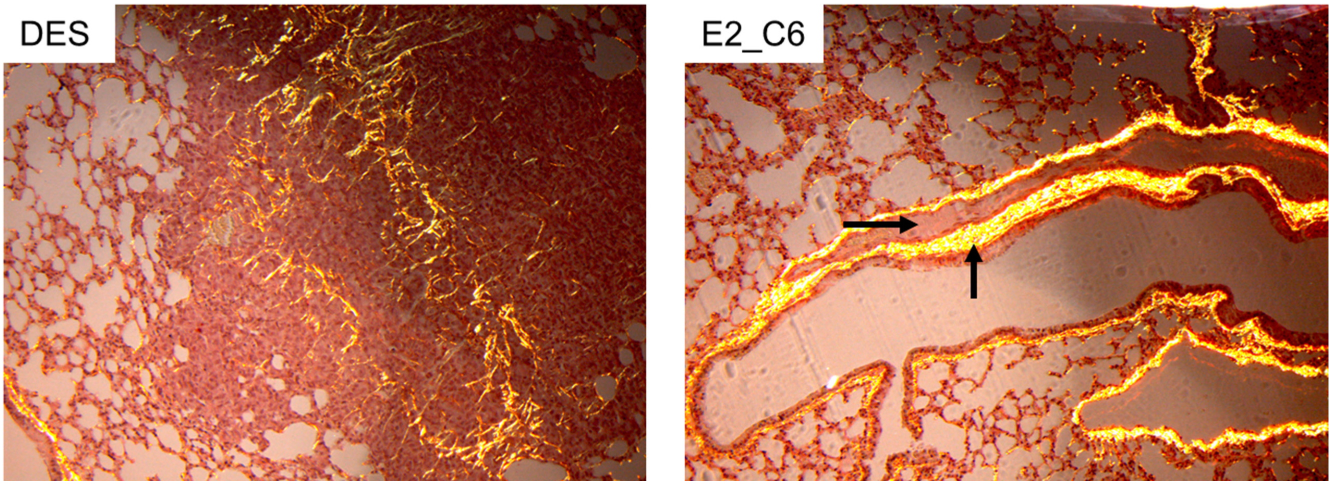

Supplementary Figure S7: Lung tissue of Fc-scFv-treated mice stained for collagen type I. The lungs of mice treated with anti-MT1-MMP E2_C6 or DES control Fc-scFv's were stained with Sirius Red to observe collagen type I. Control Fc-scFv DES-treated mice contained large metastasis with blood vessels showing thinned collagen layer. E2_C6 treated mice exhibited normal sized collagen layers (vertical arrow) surrounding blood vessels (horizontal arrow).
